# Taxonomic innovations in South American *Selaginella* (Selaginellaceae, Lycopodiophyta): description of five new species and an additional range extension

**DOI:** 10.3897/phytokeys.159.55330

**Published:** 2020-09-04

**Authors:** Iván A. Valdespino

**Affiliations:** 1 Departamento de Botánica, Facultad de Ciencias Naturales, Exactas y Tecnología, Universidad de Panamá, Apartado Postal 0824-00073, Panama Universidad de Panamá Panama Panama; 2 Sistema Nacional de Investigación (SNI), SENACYT, Panama Sistema Nacional de Investigación Panama Panama

**Keywords:** Guiana Highlands, heteromorphic, New World, non-articulate stems, South America, sporophylls, strobili, Escudo Guyanés, esporófilos, estróbilos, heteromórficas, Nuevo Mundo, Sudamérica, tallos no articulados

## Abstract

Five *Selaginella* species (i.e, *S. gioiae*, *S. papillosa*, *S. pubimarginata*, *S. rostrata*, and *S. xanthoneura*) from Neotropical rainforests of South America are described and illustrated as new, while *S. surucucusensis*, originally recorded only from Brazil, is redefined to account for species’ morphological characters throughout its expanded distribution range and also a novel illustration is provided for it. Inferred taxonomic affinities and conservation assessment are offered for species here treated. *Selaginella gioiae* is native to Colombia, Ecuador, and Peru, and *S. xanthoneura* is so far only known in Colombia, whereas *S. surucucusensis* is now known to occur in Colombia and Venezuela in the north-central part of South America. These three species are included in the “*Selaginella flabellata* group” based on their habit, stem shape, rhizophores position, and mega- and microspores color, and ornamentation. *Selaginella papillosa*, *S. pubimarginata*, and *S. rostrata* are native to Venezuela. *Selaginella papillosa* and *S. pubimarginata* morphologically belong in the “*Selaginella deltoides* group” based on their habit, stem type, shape of lateral leaves and their indument type distributed on upper surface of the leaf lamina. On the other hand, *S. rostrata* is considered to be a member of the “*Selaginella microdonta* group,” which is centered in the Guiana Highlands, based on its habit, stem type, and leaf size and shape, and for which a key to identify species is provided. Finally, all species threated here are classified in subg. Stachygynandrum based on their heteromorphic leaves, mostly quadrangular strobili, and monomorphic sporophylls shape (except for *S. rostrata* that has slightly dorsiventral and flattened strobili with somewhat heteromorphic sporophylls).

## Abstract (es)

Se describen e ilustran cinco nuevas especies de *Selaginella* (i.e, *S. gioiae*, *S. rostrata*, *S. papillosa*, *S. pubimarginata*, and *S. xanthoneura*) de los bosques neotropicales de Suramérica, mientras que *S. surucucusensis*, originalmente descrita de Brasil, es redefinida para incorporar los caracteres morfológicos de la especie a lo largo de su rango de distribución expandido y también una nueva ilustración se provee para ella. Además, se incorporan discusiones sobre las afinidades taxonómicas y el estado de conservación de las especies aquí tratadas. *Selaginella gioiae* es nativa de Colombia, Ecuador y Perú, y *S. xanthoneura* se conoce actualmente sólo de Colombia, mientras que *S. surucucusensis* ahora se reporta también para Colombia y Venezuela, en la región Norcentral de Suramérica. Estas tres especies se incluyen dentro del “grupo de *Selaginella flabellata*” debido a su hábito, tipo de tallo, posición de los rizóforos y color y ornamentación de las mega- y micrósporas. *Selaginella papillosa*, *S. pubimarginata* y *S. rostrata* se conocen sólo de Venezuela. *Selaginella papillosa* y *S. pubimarginata* pertenecen morfológicamente dentro del “grupo de *Selaginella deltoides*”, en función de su hábito, tipo de tallo, forma de las hojas laterales y la clase y distribución de su indumento en la superficie superior de la lámina. Por otra parte, *S. rostrata* pertenece al “grupo de *Selaginella nicrodonta*”, el cual se centra en el Escudo Guyanés y se caracteriza en función del hábito, tipo de tallo y forma y tamaño de las hojas de sus especies, las cuales pueden ser identificados mediante la clave que se provee. Finalmente, todas las especies aquí tratadas son clasificadas en el subg. Stachygynandrum, ya que poseen hojas heteromórficas, estróbilos cuadrangulares y esporofilos monomórficos (con la excepción de *S. rostrata*, la cual posee estróbilos ligeramente dorsiventrales y aplanados con esporofilos levemente heteromórficos).

## Introduction

*Selaginella*, a highly diverse and species rich lycophyte genus (600–800 spp), is found in a wide variety of ecosystems almost worldwide (Valdespino 2015; Zhou and Zhang 2015; PPG I 2016; Valdespino 2016; Valdespino 2017a). *Selaginella* species can be classified using several subgeneric classification systems as discussed in Valdespino and López (2019). In the New World, there are some 350 described *Selaginella* species, most of them in Neotropical ecosystems, and the number of species described from this region has steadily increased during the last decades. The latter is the result of contemporary field studies that have yielded additional collections, critical revision of herbarium material incorporating detailed microscopic analyses, and overall reassessment of previously known taxa. This tendency will most likely continue, particularly as local and regional taxonomic monographic studies and phylogenetic analysis using molecular data of infrageneric groups are undertaken. The aim of the present contribution is to further document *Selaginella* diversity in South America by describing five new species and revising the description and distribution range of another species described not long ago from this region.

## Material and methods

I studied gross morphological features of leaves and spores of *Selaginella* species using herbarium specimens from AAU, B, BM, COL, CR, F, GH, HUA, INPA, K, MG, MO, NY, PMA, R, RB, S, U, UC, US, and VEN (acronyms according to Thiers 2020) using stereomicroscopes (i.e., Olympus SZ 60-STS at the New York Botanical Garden and Olympus SZX16 at the University of Panama herbaria). I further studied fine surface details of leaves and spores using a Zeiss Model Evo 40vp Scanning Electron Microscope (SEM) at 10–15 kV at the Smithsonian Tropical Research Institute (STRI). Digitized SEM images of plant sections, leaf, and spores were taken at different magnifications, post-processed, and assembled in multipart figures using Adobe Photoshop as explained in Valdespino (2016). Terminology, measurements, and conservation status used in species descriptions follow Valdespino and López (2019) and references therein, while species classification is according to Weststrand and Korall (2016).

## Results

I describe the five new *Selaginella* species *S. gioiae* Valdespino, *S. papillosa* Valdespino, *S. pubimarginata* Valdespino, *S. rostrata* Valdespino, and *S. xanthoneura* Valdespino, and provide an updated description and an extended distribution range for *S. surucucusensis* L.A. Goés & E.L.M. Assis. All these taxa are native to Neotropical rainforest ecosystems of South America where *Selaginella* is notably diverse and species rich. *Selaginella gioiae* is known to occur in Colombia, Ecuador, and Peru, whereas *S. papillosa*, *S. pubimarginata*, and *S. rostrata* are native to Venezuela, and *S. xanthoneura* is so far only known to occur in Colombia. In addition, *S. surucucusensis*, originally described based on scanty material from Brazil, is now shown to be a more widely distributed species with an extended geographical rage in the north-central part of South America, with specimens documenting its occurrence in Colombia and Venezuela.

All species here examined have heteromorphic vegetative leaves (at least on frond-like, aerial parts of erect species), non-articulate stems, and mostly quadrangular strobili comprised by monomorphic sporophylls (except for *S. rostrata* where strobili are slightly flattened and dorsiventral, composed by somewhat dimorphic sporophylls). Therefore, I consider them to belong in the broadly defined subgenus Stachygynandrum (P. Beauv. ex Mirb.) Baker.

*Selaginella gioiae*, *S. surucucusensis*, and *S. xanthoneura* are morphologically related to the Neotropical “*Selaginella flabellata* (L.) Spring group” as defined by Hieronymus (1901: 682) and Valdespino (2017a). This group comprises some thirty-five species characterized by their fern-like habit, erect stems with leaves seemingly monomorphic before branches, as well as axillary, ventral, dorsal, and occasionally, seemingly lateral rhizophores. This group is additionally defined by white to off-white megaspores with distal faces usually reticulate or rugulate-reticulate, with each reticulum open or closed, and with echinate and perforate microstructures. It also possesses orange to pale orange microspores with distal faces capitate to baculate, and with echinate to perforate microstructures (Valdespino 2017a). The dorsal position of rhizophores and overall echinate microspore microstructure are not unique characters to this group (Valdespino 2017a). Nonetheless, what is revealing about the “*Selaginella flabellata* group” is the occurrence of the three types of rhizophores on a single stem and the degree and density in which the echinate microstructure occur in capitate to baculate projections of distal faces and in the overall surface of microspores.

*Selaginella papillosa* and *S. pubimarginata*, in turn, are morphologically similar to taxa in the “*Selaginella deltoides* A. Braun group” (Valdespino 2016) also from South America. Most species in this group (i.e., *S. aculeatifolia* Valdespino, *S. albolineata* A.R. Sm., *S. brevifolia* Baker, *S. deltoides*, *S. papillosa*, *S. pubimarginata*, and *S. sandwithii* Alston) typically have a moss-like habit, creeping stems, lateral leaves broadly ovate to ovate-deltate with upper surfaces hispidulous, each hair short and resembling prickles, tooth-like or papilla-like projections, which are mostly found submarginally, marginally, and apically (except in *S. albolineata* and *S. papillosa*) along the basiscopic halves of the laminae, and conspicuous (less so on *S. albolineata*, *S. papillosa*, and *S. pubimarginata*), straw-colored midribs. Remarkably, in the case of *S. albolineata*, the lateral leaf upper surfaces are completely covered by elongate idioblasts, while idioblasts may also be present on lower leaf surfaces of other species in this group, as well as conspicuously hyaline median leaf margins.

Lastly, *S. rostrata* is morphologically akin to species in the “*Selaginella microdonta* A.C. Smith group”, which is an alliance formed by species mainly found in the tepuis of the Guiana Highlands, particularly of Venezuela. This species group includes *S. breweriana* A.R. Sm., *S. cardiophylla* Valdespino, *S. hemicardia* Valdespino, *S. microdonta* (also found in Brazil), *S. neblinae* A.R. Sm., and *S. valdepilosa* Baker (also from Guyana). This group is characterized by its ribbon-like or leafy liverwort- to moss-like habit, creeping stems, small leaves, and usually broadly ovate-elliptic to broadly elliptic lateral leaves. Based on overall plant appearance, species of this group are commonly mistaken for bryophytes. Nonetheless, this is quickly dispelled as one observes their root-bearing rhizophores, found at branch forks throughout stems, heteromorphic leaves, which include two rows each of large, lateral and small, median leaves, as well as axillary leaves along stems. Furthermore, a key to identify species in the “*Selaginella microdonta* group” is provided.

### Taxonomic treatment

#### Selaginella gioiae

Taxon classificationPlantaeSelaginellalesSelaginellaceae

Valdespino sp. nov.

9D954FDE-AA50-5EC4-BEC2-C39705CEDB93

urn:lsid:ipni.org:names:77211382-1

**Figure 1.**
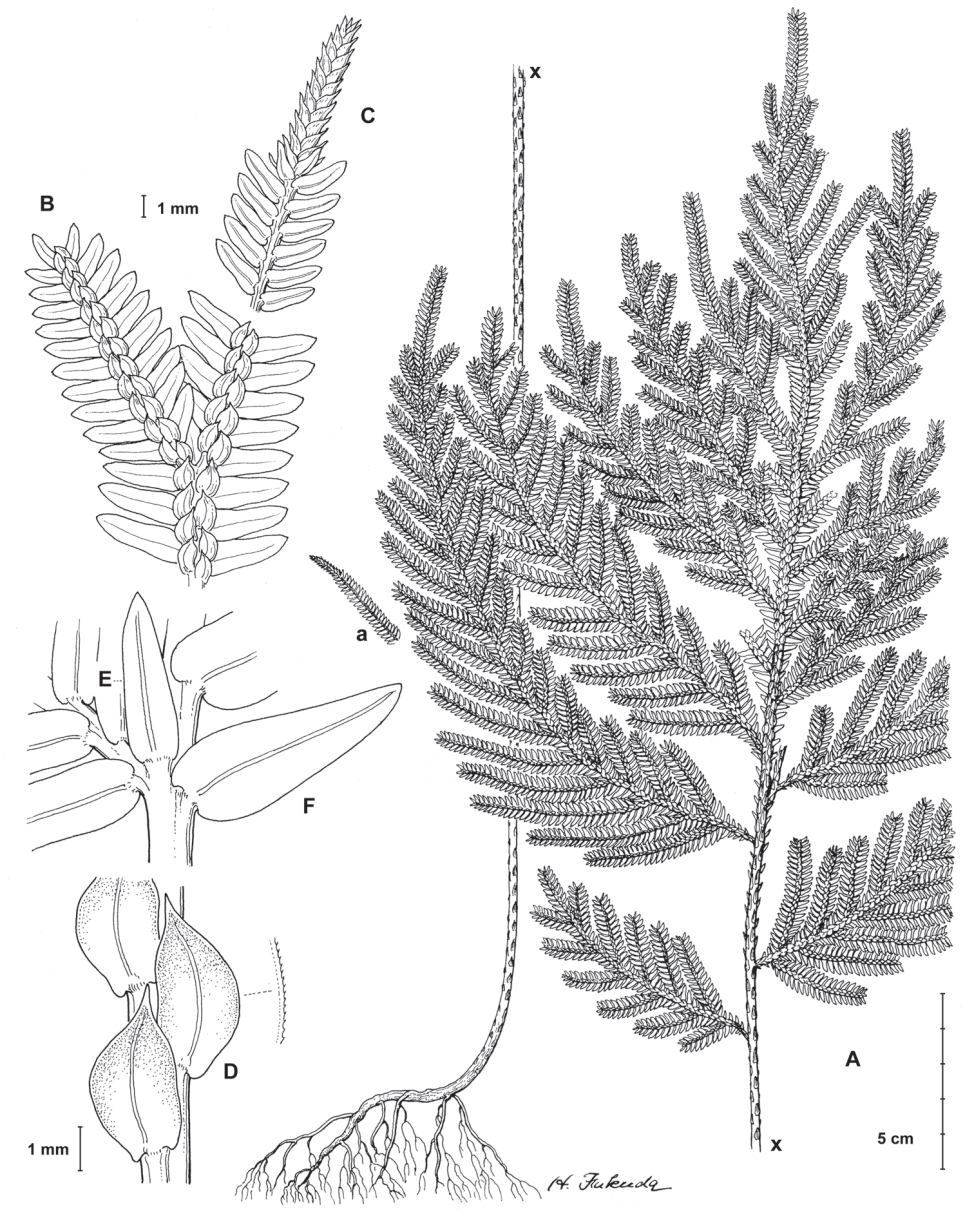
*Selaginella gioiae* Valdespino. **A** Habit, upper surface of stem and (a) detail of terminal strobili on branch, lower surface **B** branch section, upper surface **C** branch section showing terminal strobilus, lower surface **D** median leaves with details of a leaf margin, upper surface **E, F** branch section showing axillary leaf (**E**) and lateral leaf (**F**), lower surface. **A–F** line drawing made from the holotype. Illustration by Haruto Fukuda.

**Figure 2.**
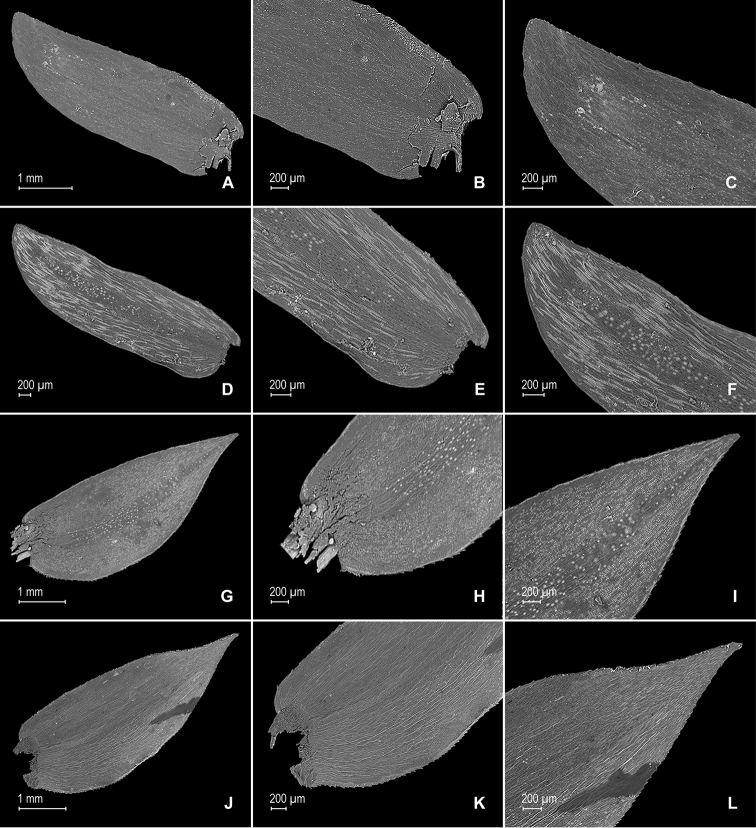
*Selaginella gioiae* Valdespino. **A** Lateral leaf from stem branch, upper surface **B** proximal half of lateral leaf, upper surface (same leaf shown in **A**) **C** distal half of lateral leaf, upper surface (same leaf shown in **A**) **D** lateral leaf, lower surface **E** proximal half of lateral leaf, lower surface (same leaf shown in **D**) **F** distal half of lateral leaf, lower surface (same leaf shown in **D**); note, elongate and papillate idioblasts (a) and stomata along midrib (b) **G** median leaf from stem branch, upper surface **H** proximal half of lateral leaf, upper surface (same leaf shown in **G**) **I** distal half of median leaf, upper surface (same leaf shown in **G**); note, elongate and papillate idioblasts (a) and stomata along midrib (b) **J** median leaf from stem branch, lower surface **K** proximal half of median leaf, lower surface (same leaf shown in **J**) **L** distal half of median leaf, lower surface (same leaf shown in **J**). **A–L** taken from the holotype.

**Figure 3.**
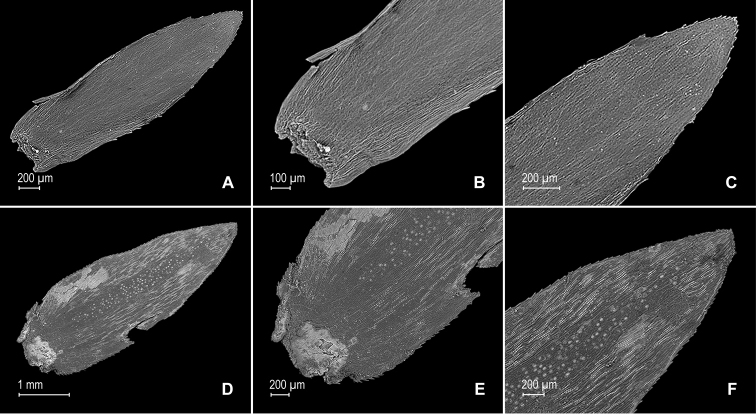
*Selaginella gioiae* Valdespino. **A** Axillary leaf from stem branch, upper surface **B** proximal half of axillary leaf, upper surface (same leaf shown in **A**) **C** distal half of axillary leaf, upper surface (same leaf shown in **A**) **D** axillary leaf from stem branch, lower surface **E** proximal half of axillary leaf, lower surface (same leaf shown in **D**) **F** distal half of axillary leaf, lower surface (same leaf shown in **D**); note, elongate and papillate idioblasts (a) and stomata along midrib (b). **A–F** taken from the holotype.

**Figure 4.**
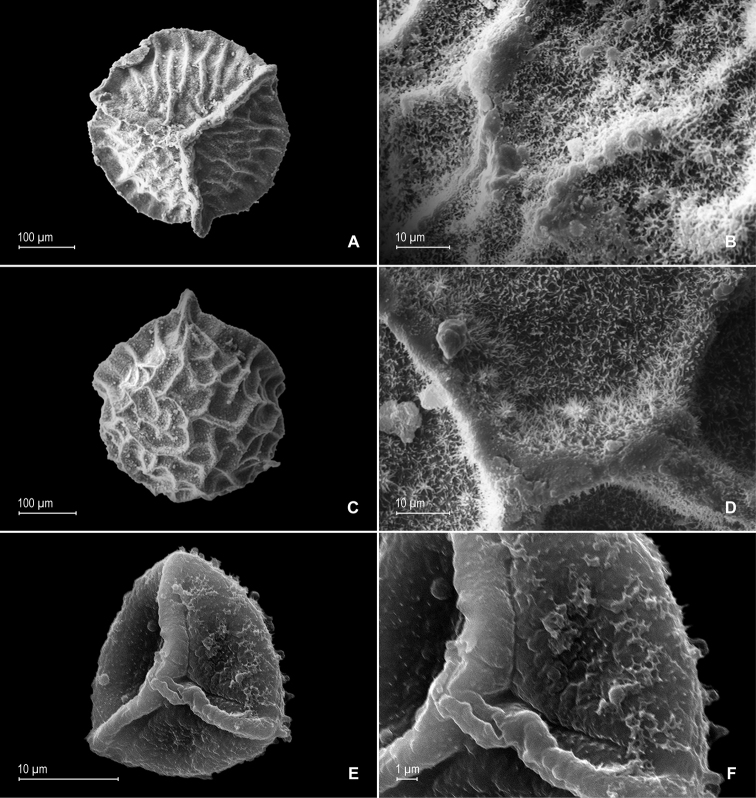
*Selaginella gioiae* Valdespino. **A** Megaspore, proximal face **B** detail of megaspore, proximal face **C** megaspore, distal face, equatorial view **D** detail of megaspore, distal face **E** microspore, proximal face **F** detail of microspore, proximal face. **A–F** taken from the holotype.

**Figure 5.**
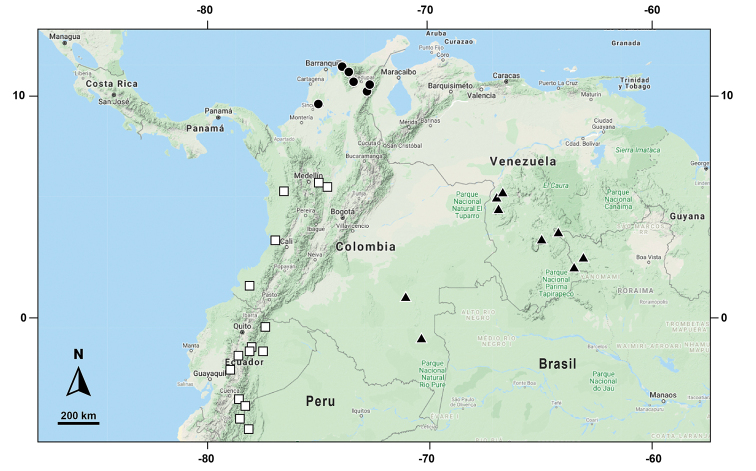
Distribution of *Selaginella gioiae* □, *S. surucucusensis* ▲, *S. xanthoneura* ●.

Figures 1 , 2 , 3 , 4 , 5

##### Diagnosis.

*Selaginella gioiae* differs from *S. surucucusensis* by the leaves on main stems before becoming fully heteromorphic, triangular-lanceolate, triangular-ovate or deltate (vs. ovate or broadly ovate), the leaves shortly before or after fourth to sixth or even further up along (vs. above first or second) stem branches fully heteromorphic and at this point onward lateral leaves oblong or oblong-ovate (vs. ovate or ovate-oblong), median leaf inner margins straight (vs. convex), and linear-lanceolate to lanceolate (vs. ovate to ovate-lanceolate) axillary leaves.

##### Type.

Colombia. Antioquia: Mpio. San Luis, 16 km SW de las partidas a San Luis, vía Medellín-Bogotá, Vereda La Josefina, 06°00'N, 74°50'W, 800 m, 25 Jun 1987 (fe), *R. Callejas et al. 4180* (holotype: NY!; isotypes: HUA!, MO!, PMA!).

##### Description.

*Plants* terrestrial. *Stems* erect, stramineous, 0.6–1.2 m tall, (2.0)2.5–6.0 mm diam. on main stem before first branches, non-articulate, not flagelliform, stoloniferous, 2 or 3-branched, the terminal portion of the stem similar in shape to lateral branches (i.e., conform). *Rhizophores* axillary, ventral, dorsal, and seemingly lateral, borne on lower-most part of the stems and throughout stolons, stout, 0.3–2.0 mm diam. *Leaves* seemingly monomorphic and strongly appressed to the stem shortly before or after fourth to sixth or even further up along stem branches (depending on stem length), then heteromorphic (of three kinds of leaves: median, lateral, and axillar), coriaceous, upper surface dull to shiny green, striate or striate-corrugate, lower surface shiny yellowish green to silvery green, striate, those on main stem before fully heteromorphic triangular-lanceolate, triangular-ovate or deltate, the bases prominently raised and truncate with both edges rounded or slightly subcordate and glabrous, the margins narrowly hyaline and denticulate, the apices attenuate. *Lateral leaves* on main stems after leaves become fully heteromorphic, distant, ascending to spreading, oblong or oblong-ovate, 2.0–4.2(5.0) × 0.9–2.2(2.5) mm; bases truncate at central portion, glabrous, acroscopic bases strongly overlapping stems, rounded, entire, basiscopic bases free from stems, geniculate; margins on upper surfaces bordered by greenish, rectangular, and laevigate cells, acroscopic margins on lower surfaces narrowly bordered continuously by a hyaline band comprised of idioblasts, the band 1–3 cells wide, the idioblasts elongate, straight-walled, and papillate, the papillae in a single row over each cell lumen, basiscopic margins on lower surfaces bordered continuously by greenish, elongate, straight-walled, laevigate cells, acroscopic margins entire to sparingly denticulate along proximal ⅔, otherwise denticulate distally, basiscopic margins entire or scarcely denticulate; apices obtuse, entire or obscurely denticulate; upper surfaces consisting of irregularly shaped, somewhat rectangular, straight to sinuate-walled cells (often difficult to distinguish because of waxy deposits), with some of these sparse- and obscurely papillate, papillae in one row on each cell lumen, without stomata or with few, obscure submarginal stomata, sparsely distributed along basiscopic margins, lower surfaces consisting of elongate, sinuate-walled cells and of elongate, straight-walled, papillate idioblasts, papillae 6–22 in one rows on each cell lumen, with stomata on 3–7 rows along central most portion of midribs. *Median leaves* on main stem after leaves fully heteromorphic, distant to slightly imbricate, ascending, ovate to ovate-lanceolate, 1.4–3.4 × 0.8–1.7 mm; bases glabrous, truncate to truncate-oblique, without auricles or the outer bases with a rounded nob; margins bordered continuously by a narrow hyaline band comprised of idioblasts, the band 1–3 cells wide, the idioblasts similar to those in acroscopic, hyaline marginal bands of lateral leaves, lower surfaces, the inner margins, straight, entire throughout or entire along proximal ½ and sparsely denticulate on distal ½, the outer margins convex, denticulate throughout; apices acute or attenuate, each 0.1–0.5 mm long, entire at tip or tipped by 1–3 small teeth; upper surfaces similar to those on upper surfaces of lateral leaves but more abundantly covered by irregularly arranged, papillate idioblasts, the papillae 3–14 in one row on each cell lumen, with stomata in 3–7 rows along midribs and few submarginal, along basiscopic ⅓ of outer margins, lower surfaces comprising elongate (somewhat jigsaw puzzle-like), sinuate-walled cells, without idioblasts and stomata. *Axillary leaves* on main stem after leaves fully heteromorphic linear-lanceolate to lanceolate, 2.5–4.5 × 1.0–1.7 mm; bases truncate, prominently raised, glabrous; margins as in lateral leaves, denticulate throughout; apices gradually tapering, broadly acute, tipped by 1–3 teeth; both surfaces as in lateral leaves. *Strobili* terminal on main stem and each branch tips, quadrangular, 0.5–5 cm long. *Sporophylls* monomorphic, without a laminar flap, each with a well-developed and glabrous keel along midribs, ovate-lanceolate, 1.5–2.0 × 0.6–1.0 mm; bases rounded to truncate; margins narrowly hyaline, 1 or 2 cells wide with the cells elongate, slightly sinuate-walled and glabrous, parallel to margins, denticulate throughout; apices attenuate to acuminate, the acumen 0.1–0.3 mm, tipped by 1–3 small teeth; *dorsal sporophylls* with upper and lower surfaces as in vegetative leaves; *ventral sporophylls* with both surfaces, silvery green to hyaline, comprised of elongate, papillate, sinuate-walled cells and of papillate idioblasts. *Megasporangia* intermixed with microsporangia along two ventral rows; *megaspores* white to beige colored, 325–350 µm diam., proximal faces rugulate with a strongly developed equatorial flange, the microstructure echinate to slightly granular, distal faces reticulate, the reticulae open (incomplete) to closed and the microstructure echinate and perforate. *Microsporangia* in two dorsal rows and intermixed with megasporangia along two ventral rows; *microspores* light orange, 18–20 µm diam., proximal faces rugulate-echinate on proximal faces with slightly punctate or rugulate microstructure, distal faces capitate or baculate, with each caput or bacula and the microstructure echinate or rugulate.

##### Habitat and distribution.

*Selaginella gioiae* grows on lowland and montane rainforests at 80–1480 m. It is known from tropical rainforest ecosystems on both sides of northwestern Andes and in the Amazon basin, specifically in western Colombia and central-eastern Ecuador and northwestern Peru. It has been collected in fertile condition from February to December.

##### Eponomy.

This unique, tall *Selaginella* species is named after Christopher Gioia (1968–), who as my longtime companion has steadfastly encouraged my work on the genus.

##### Conservation status.

*Selaginella gioiae* is a widely distributed species that grows at low and high elevations with collections continuously made from the late nineteenth- to early in the twenty-first century over a relatively wide range in South America, which suggest it is comprised of healthy populations. Accordingly, it is here considered of Least Concern (LC) based on IUCN (2012).

##### Additional specimens examined (paratypes).

Colombia. **Antioquia**: Mpio. San Luis, Autopista Medellín-Bogotá, Vereda La Josefina, road to Tulipán, caño La Mariola, 800 m, 18–19 Feb 1984, *Hoyos & Hernández 925* (MO); Río Guatapé, 3800 ft [ca. 1158 m], 23 Feb 1880, *Kalbreyer 1434* (B-4 sheets). **Chocó**: Mpio. Quibdó, Corr. Guayabal, Río Hugón, 14 Oct 1985, *García et al. 55* (COL). **Nariño**: Barbacoas, s.d., *Triana s.n.* (B), along road between Junín and Barbacoas, 1.9 km NE of Junín, 01°21'S, 78°06'W, 1300 m, 27 Feb 1992, *Croat 72431* (MO). **Valle de Cauca**: Costa del Pacífico, Río Raposo, 20–50 m, 26 Mar 1963, *Idrobo 5255* (COL); Cordoba, Dagua Valley, 80–100 m, 6–8 May 1922, *Killip 5092* (GH). Ecuador. **Morona-Santiago**: road between Gualaquiza and Indanza, 12 km S of Indanza along river, 03°11'47"S, 78°33'06"W, 1250 m, 8 Sep 2002, *Croat 87275* (MO-2 sheets); Ridge between ríos Ontza and Chupiasa, 02°40”S, 78°W, 4300–4700 ft [1311–1433 m], 17 Nov–5 Dec 1944, *Camp E-1194* (NY). **Napo**: El Chaco, Río Granadillo, Campamento de INECEL, Codo Alto, 00°08'S, 77°28'W, 1300 m, 13–15 Sep 1990, *Palacios 5783* (MO, UC); Reserva Biológica Jatun Sacha, Río Napo, 8 km abajo de Misahuallí, 01°04'S, 77°36'W, 450 m, 17 Jan–6 Feb 1987, *Cerón 748* (AAU, MO, UC). **Napo-Pastaza**: Mera, near Mangayacu, [ca. 01°42'27"S, 78°52'23"W], ca. 1100 m, *Asplund 19100* (S). **Pastaza**: El Porvenir, ca. 5 km N of Puyopungo, 17 Nov 1976, *Lugo 4897B* (BM); Mera, 1100 m, 25 May–6 Jun 1968, *Harling et al. 9779* (BM), *10128* (BM), between Puyo and Baños, ca. 5 km W of Mera, 01°26'S, 78°08'W, 1100 m, 7 Mar 1992, *Croat 72833* (MO); Near Napo road, 9 km N of Puyo, 18 Apr 1958, *Prescott 1361* (NY); Hacienda San Antonio de Barón von Humboldt, 2 km NE de Mera, 01°27'S, 78°06'W, 1100 m, 20 Feb–20 Mar 1985, *Palacios et al. 23A* (AAU, MO, NY), *Zaruma et al. 6* (AAU, MO, NY, UC); Vicinity of Shell, 1.6 km N of main Baños-Puyo road, along Río Claro, 40°29'39"S, 78°03'52"W, 1085 m, 9 Oct 2007, *Croat et al. 99520* (MO). **Tungurahua**: ca. 5 km E of town of Río Negro, 1350 m, 17 Jan 1973, *Humbles 6120* (MO). **Zamora-Chinchipe**: Cordillera del Condor, Chinapintza, trail to Destacamento Mayaycu Alto, 04°03'S, 78°35'W, 1350–1480 m, 6 Dec 1990, *Øllgaard 98418* (AAU); Vicinity of mining camp at Río Tundaime, along Río Quimi, 03°31'10"S, 78°25'53"W, 900–1000 m, 3 Nov 2004, *van der Werff et al. 19255* (MO, NY). Peru. **Amazonas**: Dist. El Cenepa, Tutino, Quebrada Tutino, 04°34'31"S, 78°11'34"W, 300 m, 22 Jul 1997, *Rojas et al. 136* (MO, NY).

##### Discussion.

*Selaginella gioiae* is characterized by its fern-like habit, erect stems, each 0.6–1.2 m tall, leaves on main stem normally fully heteromorphic shortly before or after fourth to sixth or even further shortly before or after fourth to sixth or at higher up branches depending on main stem length, with margins bordered by a narrow band of hyaline, papillate, idioblasts. On main stems it has oblong or oblong-ovate lateral leaves, median leaf bases glabrous, truncate to truncate-oblique, without auricles or the outer bases rounded or with a rounded nob, linear-lanceolate or narrowly lanceolate axillary leaves, megaspores with a prominent equatorial flange, and microspores distal faces capitate or baculate, each caput or baculum usually micro-echinate. *Selaginella gioiae* is further notable by its median leaf lower surfaces with frequently very well defined or marked midribs and strobili tips occasionally displaying vegetative growth.

*Selaginella gioiae* is one of the tallest species within the “*Selaginella flabellata* group” and among these taxa it may be confused with *S. surucucusensis* because of their fairly similar median leaves with inconspicuous, short-elongate or punctate idioblasts on the upper surfaces. *Selaginella gioiae*, however, is set aside from *S. surucucusensis* by the characters listed in the diagnosis and by its median leaf with the outer bases rounded or with a rounded nob (vs. with a distinct auricle) and acute, attenuate or short-acuminate (vs. attenuate or acuminate to short-aristate) apices, each less than ¼ (vs. ¼) the length of the lamina. *Selaginella cuneata* Mickel & Beitel is another member of the “*Selaginella flabellata* group” with inconspicuous short-elongate or punctate idioblasts on the upper surface of median leaves. *Selaginella gioiae* differs from the latter by its median leaf outer half of the lamina at least ¼ to ½ wider (vs. twice as narrow) as the inner half, and hyaline (vs. greenish) bordered margins of median leaves and on acroscopic margins of lateral leaves.

#### Selaginella papillosa

Taxon classificationPlantaeSelaginellalesSelaginellaceae

Valdespino sp. nov.

C006952C-1005-5AD0-A03E-CDC544AF6E88

urn:lsid:ipni.org:names:77211383-1

**Figure 6.**
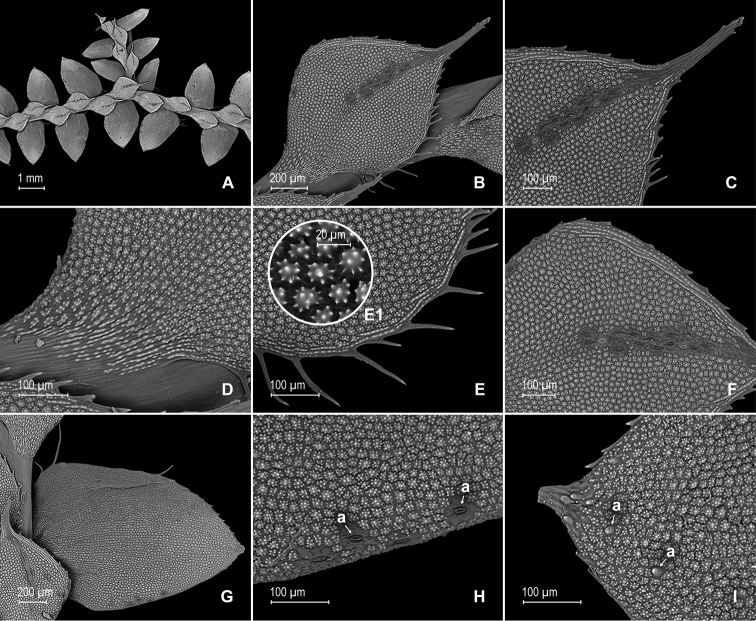
*Selaginella papillosa* Valdespino. **A** Section of upper surface of stem showing median and lateral leaves **B** median leaf, upper surface **C** distal half of median leaf, upper surface (same leaf shown in **B**) **D** proximal half of median leaf, upper surface (same leaf shown in **B**) **E** section median leaf inner half, upper surface; note prominent papillae on cells (E1) **F** detail of midsection of median leaf, upper surface **G** lateral leaf and sections of median leaves, upper surface **H** portion of basiscopic margin of lateral leaf, upper surface; note submarginal stomata (a) **I** distal half of lateral leaf, upper surface, note short hair on leaf surface (a). **A–I** taken from the holotype.

**Figure 7.**
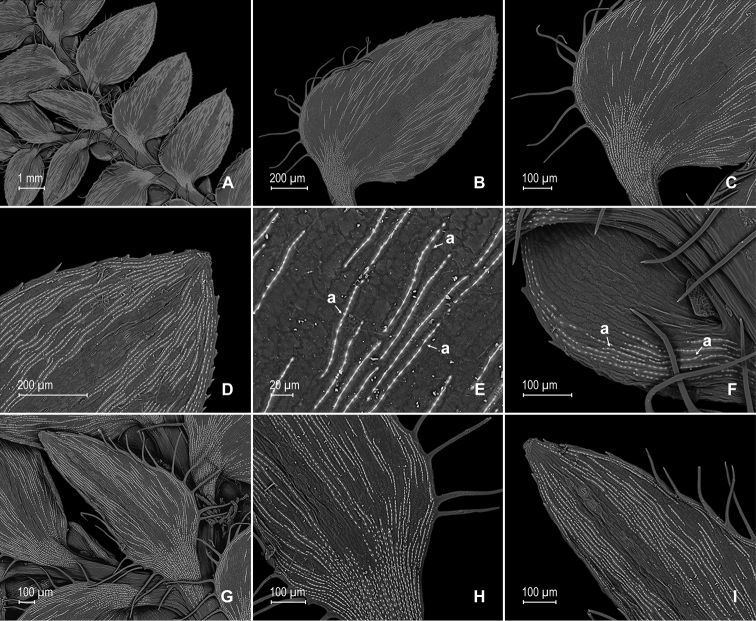
*Selaginella papillosa* Valdespino. **A** Section of lower surface of stem showing lateral leaves and axillar leaf **B** lateral leaf, lower surface **C** proximal half of lateral leaf, upper surface (same leaf shown in **B**) **D** distal half of lateral leaf, upper surface (same leaf shown in **B**) **E** detail of lateral leaf, lower surface; note elongate, papillate idioblasts with papillae interconnected (a) **F** detail of outer half of median leaf, lower surface; note elongate, papillate idioblasts (a) **G** axillary leaf and portion of lateral leaves, lower surface **H** portion of proximal half of axillary leaf, lower surface (same leaf shown in **G**) **I** distal half of axillary leaf, lower surface (same leaf shown in **G**). **A–I** taken from the holotype.

**Figure 8.**
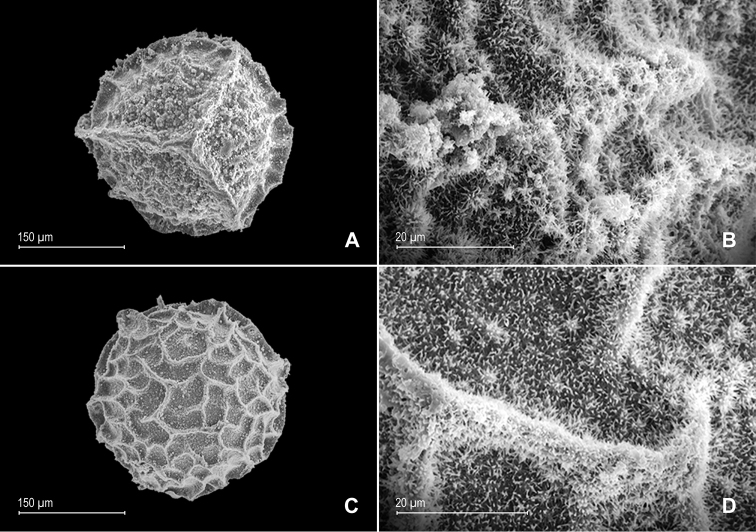
*Selaginella papillosa* Valdespino. **A** Megaspore, proximal face **B** detail of megaspore, proximal face **C** megaspore, distal face **D** detail of megaspore, distal face. **A–D** taken from the holotype.

**Figure 9.**
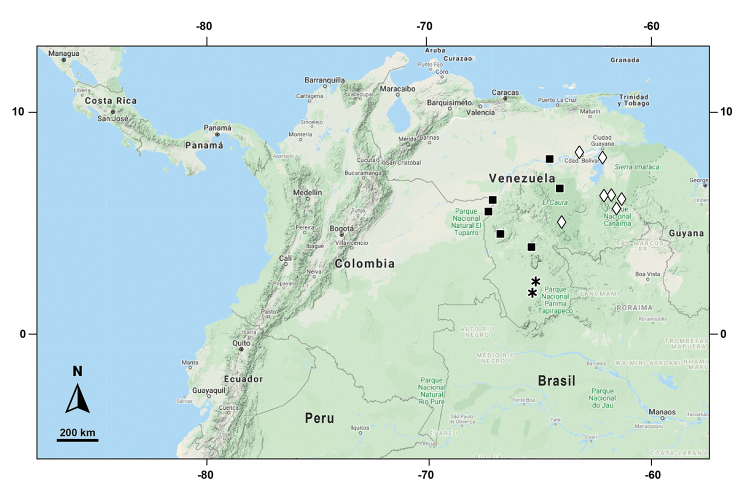
Distribution of *Selaginella papillosa* *, *S. pubimarginata* ◊, *S. rostrata* ■.

Figures 6 , 7 , 8 , 9

##### Diagnosis.

*Selaginella papillosa* differs from *S. brevifolia* by its median leaf inner and outer halves equal in width (vs. outer halves typically wider than the inner halves), lateral leaf upper surfaces with midribs not marked and of the same color as the rest of the laminae (vs. well-marked and straw-colored), acroscopic margins long-ciliate along proximal ¼−⅓ or occasionally proximal ½ (vs. ½−¾), and axillary leaf ovate or ovate-lanceolate (vs. ovate-deltate).

##### Type.

Venezuela. Amazonas: Río Negro, Río Siapa near base of Cerro Aracamuni, 01°39'N, 65°40'W, 250 m, 4 Nov 1987, *R.L. Liesner & G. Carnevali 22750* (holotype: NY!; isotypes: MO!, NY!, UC!).

##### Description.

*Plants* epipetric, moss-like. *Stems* creeping, stramineous, 5.0–15.0 cm long, 0.3–0.5 mm diam., non-articulate, not flagelliform, not stoloniferous, straw-colored, 1 or 2-branched, the branches arising at almost 90° angle. *Rhizophores* axillary, axillary-ventral or dorsal, borne throughout the stems, filiform, 0.1–0.2 mm diam. *Leaves* heteromorphic throughout, membranaceous, upper surfaces light green, lower surfaces silvery green. *Lateral leaves* distant, spreading to slightly ascending or slightly imbricate (at branch tips), ovate, 1.0–2.0 × 0.8–1.6 mm; bases rounded, glabrous, acroscopic bases overlapping stems, basiscopic bases free from stems; margins on upper surfaces greenish and composed of quadrangular to rounded cells, on lower surfaces bordered continuously by a hyaline band comprised of idioblasts, the band 1–3 cells wide, the idioblasts elongate, straight-walled, and papillate, the papillae in a single row over each cell lumen, acroscopic margins long-ciliate along proximal ½–⅔, otherwise dentate distally, basiscopic margins dentate along proximal ⅔, otherwise denticulate distally; apices acute, attenuate to apiculate, apiculae often falling off, tipped by 1 or 2 teeth; upper surfaces mostly glabrous, except for few, distal, teeth-like hairs near apices, comprising rounded to quadrangular, sinuate-walled, papillate cells, each cell lumen with 7–14 papillae, with few (ca. 4) submarginal to marginal stomata near central portion of basiscopic margins, lower surfaces glabrous, comprising elongate, sinuate-walled, laevigate cells and of straight-walled, papillate idioblasts, the papillae 9–15 in one row on each cell lumen, with stomata on 1–3 rows along midribs. *Median leaves* distant to slightly imbricate near branch tips, ascending, ovate to broadly ovate to ovate-orbiculate or elliptic with both inner and outer halves equal in width, 0.8–1.2 × 0.5–1.0 mm; bases glabrous, oblique and decurrent, without auricles; inner margins bordered continuously by a narrow hyaline band comprised of idioblasts, the band 1 or 2 cells wide, the idioblasts similar to those in the hyaline marginal bands of the lateral leaves on lower surfaces, except for papillae sometimes interconnecting, long-ciliate along proximal ¾, otherwise short-ciliate to dentate distally, the outer margins bordered by greenish, quadrangular to elongate, glabrous cells along proximal ½ and along distal ½ by a hyaline band comprised of idioblasts, the band 1–5 cells wide, the idioblast similar to those in the inner margins, dentate throughout; apices aristate, each arista 0.2–0.4 mm long, tipped by 1 or 2 teeth; upper surfaces glabrous, comprising rounded, sinuate-walled, papillate cells, each cell lumen with 4–15 papillae, concentrically arranged, without idioblasts, with stomata in 1 or 2 rows along midribs on distal ½ of the leaf lamina and few (1 or 2) marginal to submarginal along proximal ½ of outer margins, lower surfaces comprising elongate, sinuate-walled, glabrous cells and submedial to submarginal, sinuate-walled, papillate, idioblasts cells, the papillae similar to those in lower surfaces of lateral leaves, without stomata. *Axillary leaves* ovate to ovate-lanceolate, 1.2–1.5 × 0.6–1.0 mm; bases attenuate and covered by idioblasts similar to those in lower surfaces of lateral leaves, except for papillae in 1 or 2 rows on cell lumen; margins on upper and lower surfaces as in lateral leaves, ciliate along proximal ½–¾; apices acute, attenuate to apiculate, apiculae often falling off; both surfaces as in lateral leaves. *Strobili* terminal on branch tips, quadrangular, 0.4–5 mm long. *Sporophylls* monomorphic, without a laminar flap, each with a slightly developed keel along midribs, the keel glabrous or with few, short, tooth-like projections distally, ovate-lanceolate, 0.6–1.0 × 0.3–0.5 mm; bases rounded; margins narrowly bordered by a hyaline band, the band 1 or 2 cells wide with the cells elongate, slightly sinuate-walled and glabrous, shortly ciliate along proximal ½, denticulate on distal ½ or denticulate throughout; apices attenuate to acuminate, the acumen 0.1–0.3 mm long, tipped by 1 or 2 teeth; *dorsal sporophylls* with upper surfaces green and cells as in median leaves, except for the half that overlaps the ventral sporophylls where the surfaces are greenish hyaline to hyaline with elongate and slightly sinuate-walled cells, lower surfaces hyaline and comprising elongate, sinuate-walled cells; *ventral sporophylls* with both surfaces, hyaline, comprised of elongate, papillate, sinuate-walled cells. *Megasporangia* few and proximal, along two ventral rows; *megaspores* yellow, pale yellow to whitish, 270–310 µm diam., proximal faces rugulate-reticulate with a well-developed equatorial flange, the microstructure echinate and perforate, distal faces reticulate, the reticulae open (incomplete) to closed, the microstructure echinate and perforate. *Microsporangia* in two dorsal rows and distally along two ventral rows; *microspores* light orange, not measured or observed in detail.

##### Habitat and distribution.

*Selaginella papillosa* grows at 250–1670 m in tropical rainforests at the base of Cerro Aracamuni and near the summit of Cerro Aratitiyope in the state of Amazonas, Venezuela.

##### Etymology.

The specific epithet derives from the Latin “*papilla*,” meaning “nipple”, and refers to the abundant and distinctive papillae found on cells lumen in upper, leaf surfaces.

##### Conservation status.

*Selaginella papillosa* is only known from two collections made at the base of Cerro Aracamuni, a Guiana Highland sandstone tepui, part of the Serranía de La Neblina National Park, and near the top of Cerro Aratitiyope, a granitic mountain designated as a Natural Monument, both in the state of Amazonas, Venezuela. Accordingly, the natural populations of this new species may not be threatened. However, as available data is scanty, it does not allow for a reliable conservation assessment. Therefore, the species is considered Data Deficient (DD) based on IUCN (2012).

##### Additional specimens examined (paratypes).

Venezuela. **Amazonas**: Río Negro, Cerro Aratitiyope, ca. 70 km al SSW de Ocamo, 02°10'N, 65°34'W, 990–1670 m, 24–28 Feb 1984, *Steyermark et al. 130146* (MO-2 sheets, UC pp [mixed with some scraps of *S. tuberculata* Spruce ex Baker]).

##### Discussion.

*Selaginella papillosa* is distinctive by its moss-like habit, lateral leaf with long-ciliate acroscopic margins along proximal ½–⅔ and acute, attenuate to apiculate apices, with apiculae often falling off and tipped by 1 or 2 teeth, the upper surfaces glabrous, except for few, distal, teeth-like hairs near apices, and with few submarginal to marginal stomata near central portion of basiscopic margins. In addition, its leaf upper surfaces are comprised by rounded to quadrangular, sinuate-walled, papillate cells, each cell lumen with many (i.e., 7–14) papillae, and with margins bordered by elongate, straight-walled, papillate idioblasts on lower leaf surfaces. Finally, its median leaf upper surfaces have marginal to submarginal stomata along proximal ½ of outer margins.

*Selaginella papillosa* is a member of the “*Selaginella deltoides* group” as defined by Valdespino (2016). Among species in that alliance it is morphologically close (e.g. in habit, leaf, and overall megaspore sculpturing pattern) to *S. aculeatifolia* and the here described *S. pubimarginata* (see for discussion). These three species might, in addition, be sympatric, as they have been collected in the Guiana Highland region of Venezuela at similar low to mid elevations. *Selaginella papillosa* differs from *S. aculeatifolia* by its lateral leaf upper surfaces with few (vs. without) submarginal stomata along central, basiscopic portion of the laminae, with 3–5 (vs. many, ca. 50) short hairs or tooth-like projections near distal most portion of apices (vs. along distal ½ of basiscopic halves and apices), with midribs inconspicuous (vs. conspicuous, outlined by elongate, straight-walled and papillate idioblasts), and acute to apiculate (vs. long-acuminate) apices. It is further differentiated from the latter by its median leaves broadly ovate to ovate-orbiculate or elliptic (vs. broadly ovate to ovate-elliptic), with oblique and decurrent (vs. oblique or rounded) bases, and stomata present on upper leaf surfaces along distal ½ (vs. ¾) and with few (vs. without) stomata submarginally to marginally along proximal ½ portion of outer margins. Furthermore, its megaspores proximal and distal faces microstructure consists of long- (vs. short-) echinae.

*Selaginella papillosa* was confused in the past with *S. brevifolia* and, in fact, its type collection was originally identified as the latter species. *Selaginella papillosa* differs from *S. brevifolia* by the characters listed in the diagnosis and by its lateral leaf having only very few, i.e., 4–6 tooth-like short hairs near the apices, whereas the latter species has many of these hairs along the basiscopic halves of the laminae and toward the apices.

*Selaginella papillosa* is also set aside from the similar *S. albolineata* by its median and lateral leaves upper surfaces without (with many, elongate) idioblasts and acroscopic margins long-ciliate along proximal ½–⅔, otherwise denticulate distally (vs. entire to scarcely denticulate throughout), and median leaf inner margins long-ciliate (vs. denticulate).

The paratype specimen of *S. papillosa* (*Steyermark et al. 130146* at MO) was originally identified as *S. revoluta* Baker, vel aff., a species that is the center of a species group described by Mickel et al. (2004). *Selaginella papillosa* is a rather more slender species than *S. revoluta* and differs further from the latter by its median leaves ovate to broadly ovate to ovate-orbiculate or elliptic (vs. broadly ovate, ovate orbicular to ovate-rhombic) with oblique to decurrent (vs. subcordate) bases, and apices with rather narrow and needle-like (vs. broad and subulate) long aristae, each without (vs. with tooth-like) hairs on its surfaces. Finally, *S. papillosa* could also be compared to *S. hirtifolia* Valdespino, which is not morphologically close to it but rather is a member of the *S. revoluta* group. *Selaginella papillosa* is easily separated from the latter by its lateral leaf upper surfaces almost completely glabrous (vs. with short, teeth-like hairs along the basiscopic, submarginal region and near apices) and long-aristate (vs. acuminate) median leaf apices with the arista hyaline (vs. with the acumen green).

#### Selaginella pubimarginata

Taxon classificationPlantaeSelaginellalesSelaginellaceae

Valdespino sp. nov.

25D6A531-A643-5231-8DA2-5DFEE1023E9C

urn:lsid:ipni.org:names:77211384-1

**Figure 10.**
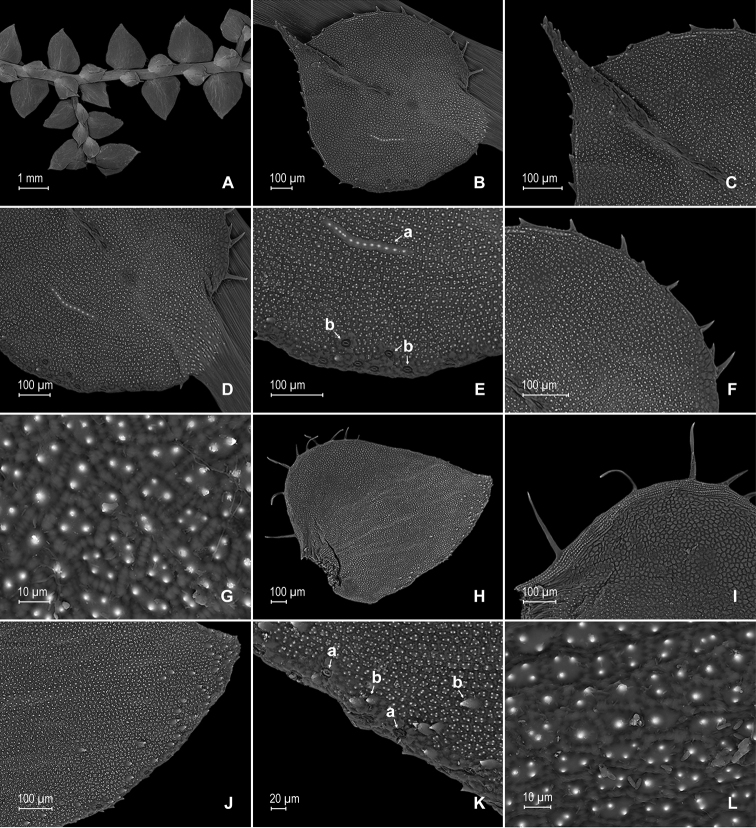
*Selaginella pubimarginata* Valdespino. **A** Section of upper surface of stem showing median and lateral leaves **B** median leaf, upper surface **C** distal half of median leaf, upper surface (same leaf shown in **B**) **D** proximal half of median leaf, upper surface (same leaf shown in **B**) **E** section of median leaf outer half, upper surface; note prominent elongate, papillate idioblast (a) and submarginal stomata (b) **F** detail of midsection of inner half of median leaf, upper surface **G** detail of median leaf, upper surface; note papillate cells **H** lateral leaf, upper surface **I** section of proximal, acroscopic half of lateral leaf, upper surface (same leaf shown in **H**) **J** section of distal and basiscopic half of lateral leaf, upper surface (same leaf shown in **H**) **K** detail of section of basiscopic margin of lateral leaf, upper surface (same leaf shown in **H**); note submarginal stomata (a) and teeth-like hairs (b) **L** detail of lateral leaf, upper surface; note papillate cells. **A–L** taken from the holotype.

**Figure 11.**
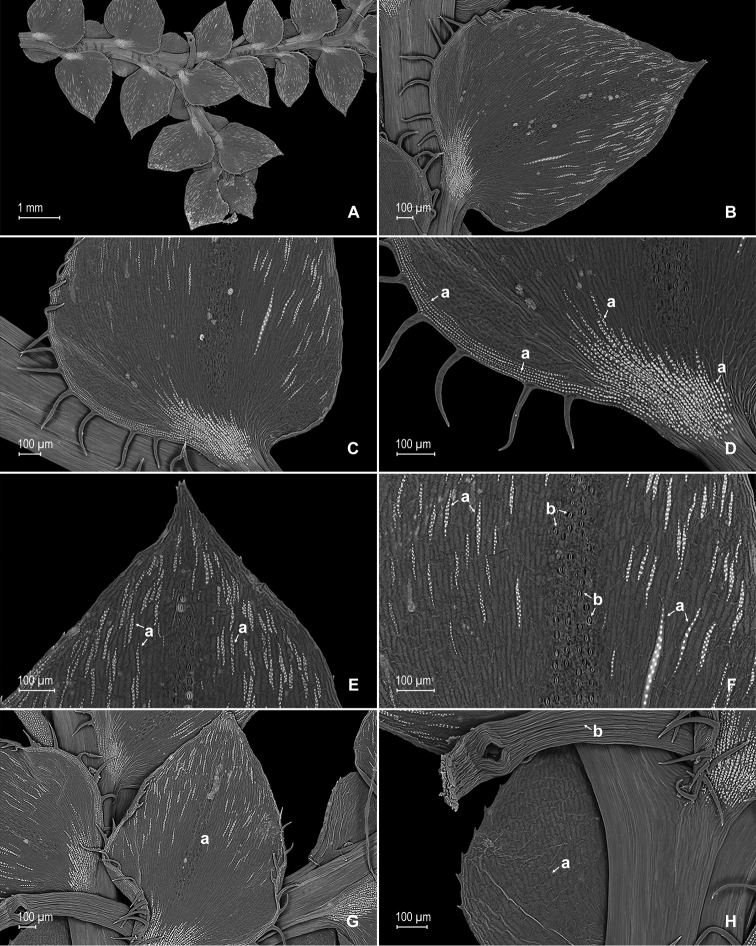
*Selaginella pubimarginata* Valdespino. **A** Section of lower surface of stem showing lateral and median leaves and axillar leaf **B** lateral leaf, lower surface **C** proximal half of lateral leaf, upper surface (same leaf shown in **B**) **D** section of proximal acroscopic margin and base of lateral leaf, lower surface (same leaf shown in **B**); note elongate, papillate idioblasts (a) **E** distal half of lateral leaf, lower surface (same leaf shown in **B**); note elongate, papillate idioblasts (a) **F** mid-section of lateral leaf, lower surface (same leaf shown in **B**); note elongate, papillate idioblasts (a) and stomata along midrib (b) **G** axillary leaf (a) and portions of lateral and median leaves, lower surfaces **H** detail of outer half of median leaf (a) and axillary rhizophores (b), lower surfaces (same leaf shown in **G**). **A–H** taken from the holotype.

**Figure 12.**
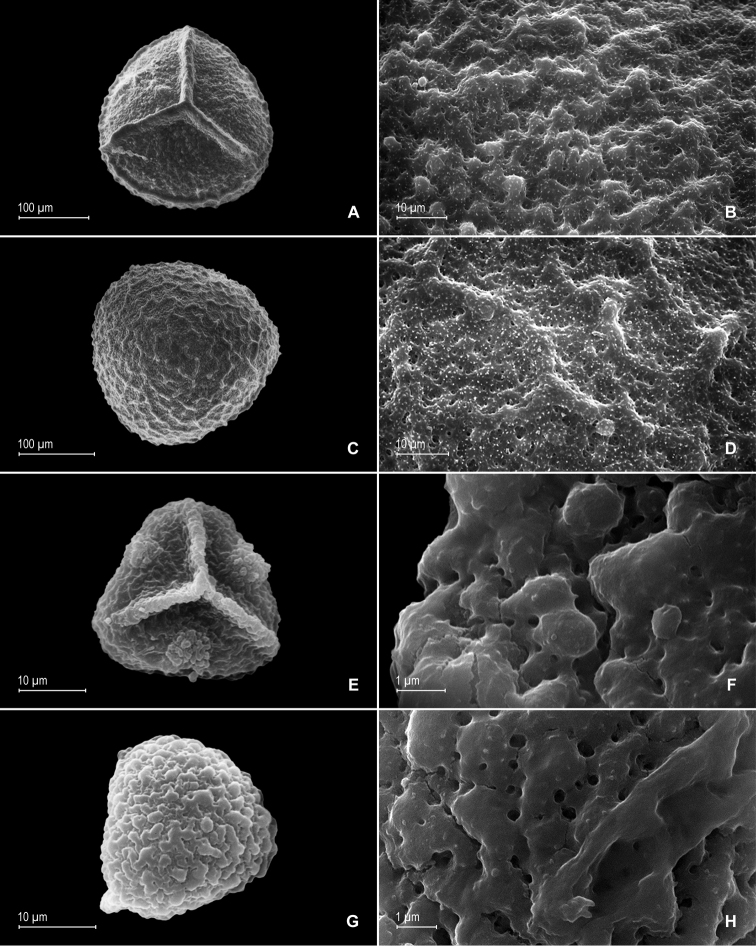
*Selaginella pubimarginata* Valdespino. **A** Megaspore, proximal face **B** detail of megaspore, proximal face **C** megaspore, distal face **D** detail of megaspore, distal face **E** microspore, proximal face **F** detail of microspore, proximal face **G** microspore, distal face **H** detail of microspore, distal face. **A–H** taken from the holotype.

Figures 9 , 10 , 11 , 12

##### Diagnosis.

*Selaginella pubimarginata* differs from *S. albolineata* by its ovate-deltate (vs. ovate to ovate-elliptic) lateral leaves with upper surfaces lacking (vs. with) conspicuous idioblasts, acroscopic margins long-ciliate along proximal ½, otherwise dentate to denticulate distally (vs. denticulate throughout), acute to attenuate to shortly acuminate (vs. obtuse to rounded) apices, and axillary leaves ciliate (vs. dentate) along proximal ½–⅔.

##### Type.

Venezuela. Amazonas: Atabapo: Río Cunucunuma, entre las comunidades de Culebra y Huachamacari, entre el Cerro Duida y Huachamacari, 180–210 m, 03°40'N, 65°45'W, 28–30 Jan & 6–8 Feb 1982, *J.A. Steyermark et al. 125655* (holotype: NY!; isotypes: MO!, NY!, PMA!, UC!).

##### Description.

*Plants* epipetric, terrestrial or epiphytic, moss-like. *Stems* creeping, stramineous, 2.0–12 cm long, 0.25–0.4 mm diam., not-articulate, not flagelliform or stoloniferous, straw-colored to brownish, 1- or 2-branched, the branches arising at ca. 45° angle. *Rhizophores* axillary, borne throughout the stems, filiform, 0.1–0.15 mm diam.

*Leaves* heteromorphic throughout, membranaceous, upper surfaces green, golden brown (in old leaves) or dark brown (in alcohol-preserved specimens), lower surfaces silvery. *Lateral leaves* distant, spreading, perpendicular to stems or less often slightly ascending, broadly ovate, ovate-deltate or ovate-elliptic, 1–1.5 × 0.5–1.2 mm; bases rounded, glabrous, acroscopic bases slightly overlapping stems, basiscopic bases free from stems; acroscopic margins on both surfaces plane or sometimes revolute on upper surfaces, along proximal ½–⅔ bordered by a hyaline band comprised of idioblasts, the band 1–6 cells wide, the idioblasts elongate, straight-walled, and papillate, the papillae in a single or double row over each cell lumen, long-ciliate, otherwise on distal ⅓–½, greenish, bordered by quadrangular to rounded cells, entire or sparse- and minutely denticulate, especially along distal ⅓, basiscopic margins on both surfaces greenish, bordered by quadrangular to rounded cells, entire throughout or sparse- and minutely denticulate, along distal ⅓; apices attenuate to shortly acuminate, each acumen ca. 0.1 mm long, tipped by 1–3 teeth; upper surfaces mostly glabrous, except for many, short, teeth-like hairs marginally to submarginally along basiscopic margins and distally towards and at the apices, comprising rounded to quadrangular, slightly sinuate, and broad-walled cells on basiscopic ⅔ of the laminae, the cells papillate, the papillae 1–5 and irregularly arranged on each cell lumen, on acroscopic ⅓ of the laminae comprising strongly sinuate, thin-walled, and glabrous cells, without idioblasts, stomata present on acroscopic margins along distal ⅓ and on basiscopic margins along distal ¾, lower surfaces glabrous, comprised of elongate, sinuate-walled, laevigate cells and of straight-walled, papillate idioblasts, the papillae 7–28 in one or two rows on each cell lumen, the idioblasts evenly distributed on the laminae and strongly grouped on proximal, basal region of the laminae, with stomata on 1–5 rows along midribs. *Median leaves* distant to slightly imbricate near branch tips, ascending, broadly elliptic to orbicular or broadly ovate-elliptic with both inner and outer halves equal in width, 0.7–1.2 × 0.5–0.8 mm; bases glabrous, oblique, not decurrent, without auricles; margins plane or outer margins on proximal ¼ revolute, along proximal ½ bordered by greenish, quadrangular cells, on distal ½ bordered continuously by a narrow hyaline band comprised of idioblasts, the band 1 or 2 cells wide, the idioblasts similar to those in the hyaline marginal bands of proximal ½–⅔ of acroscopic margins of the lateral leaves, dentate on outer margins and short-ciliate along proximal ½, otherwise dentate along distal ½ on inner margins; apices short acuminate, each acumen 0.1 or 0.2 mm long and tipped by 1–3 teeth; upper surfaces glabrous, except for few, submarginal, short, teeth-like hairs on mid portion of outer margins, comprised of round to quadrangular slightly sinuate, thick-walled, papillate cells and one, papillate idioblast along mid-section of outer margins, the papilla 10–13 on a single row on each cell lumen, stomata on midrib along distal ½ and submarginally to marginally along proximal ½ of outer margins, lower surfaces glabrous, comprised of elongate, sinuate-walled, laevigate cells, without stomata. *Axillary leaves* broadly ovate, ovate-deltate or ovate-elliptic, 1.2–1.6 × 0.7–1.2 mm; bases attenuate; margins bordered continuously by a hyaline band comprised of papillate idioblasts along proximal ⅔, the band 1–6 cells wide, the papillae in a single row, on distal ⅓ bordered by greenish, quadrangular cells, long-ciliate along proximal ½–⅔, otherwise entire to denticulate distally; apices attenuate, each 0.1 mm long, tipper by 1–5 teeth. *Strobili* terminal, loosely quadrangular, 1.5–9 mm long. *Sporophylls* monomorphic, without a laminar flap, each with a slightly developed keel along midribs, the keel puberulent with short, tooth-like projections distally, ovate to ovate-lanceolate, (0.6)0.9–1.5 × (0.3)0.5–0.7 mm; bases rounded; margins obscurely hyaline bordered by 1–3(4) elongate, papillate idioblasts (especially apically), serrate; apices long-acuminate, tipped by 1–3 teeth; *dorsal sporophylls* with upper surfaces green and cells as in median leaves, except for the half that overlaps the ventral sporophylls where the surfaces are greenish hyaline to hyaline with elongate and slightly sinuate-walled cells, lower surfaces hyaline and comprising elongate, sinuate-walled cells; *ventral sporophylls* with both surfaces, hyaline, comprised of elongate, papillate, sinuate-walled cells. *Megasporangia* few and proximal, along two ventral rows; *megaspores* light to lemon-yellow, 240–250 µm diam., proximal faces rugulate with a slightly developed equatorial flange, the microstructure sparsely, short echinate and perforate, distal faces rugulate to reticulate, the rugulae or reticulae open (incomplete) to closed, each reticulum with low walls, the microstructure sparsely, short echinate and perforate. *Microsporangia* light orange in two dorsal rows and distally along two ventral rows; 22–250 µm diam., *microspores* light orange, rugulate on proximal and distal faces, with the microstructure perforate.

##### Habitat and distribution.

*Selaginella pubimarginata* grows on shaded or exposed moist boulders, along streambeds, and waterfalls in tropical rainforests at 80–700 m; it is known at and around Cerro Huachamacari, along the rivers Sipapo and Coromoto in Amazonas state, and along Río Caura in Bolívar state, Venezuela.

##### Etymology.

The epithet derives from the Latin “*puberulus*,” slightly pubescent, and “*marginatus*,” having a border. Together these refer to the presence of submarginal hairs on lateral leaves upper surfaces near the basiscopic and apical portion of leaf laminae.

##### Conservation status.

*Selaginella pubimarginata* is known from four collections made in two states in the Guiana Highland region of Venezuela. Current available data, however, is scanty and does not allow for a reliable conservation assessment. Accordingly, this new species is considered Data Deficient (DD) based on IUCN (2012).

##### Additional specimens examined (paratypes).

Venezuela. **Amazonas**. Atabapo: Cerro Huachamacari, E slope, 03°49'N, 65°42'W, 600–700 m, 2 Nov 1988, *Liesner 25697* (MO, UC); Río Cunucunuma, entre las comunidades de Culebra y Huachamacari, entre el Cerro Duida y Huachamacari, 03°40'N, 65°45'W, 180–210 m, 28–30 Jan & 6–8 Feb 1982, *Steyermark et al. 125639* (NY, UC), 200–400 m, 28–30 Jan & 6–9 Feb 1982, *Guariglia et al. 1676* (NY-2 sheets); Atures: 125 km de la boca (delta) del Guayapo en Sipapo, 04°22'N, 67°06'W, 130 m, May 1989, *Foldats & Velazco 9203* (NY); 40 km S of Puerto Ayacucho, Tobogán de la Selva, 05°35'N, 67°30'W, 70–100 m, 21 Jan 1985, *Beitel & Buck 85010* (NY, UC); Río Coromoto, above Tobogán de la Selva, 35 km SE of Puerto Ayacucho, 05°27'N, 67°33'W, 80 m, 7 Sep 1985, *Steyermark et al. 131528* (MO, UC). **Bolívar**: alrededor del campamento “Las Pavas”, vecindad del Salto Para, Río Caura (lado derecho del río abajo), 230–280 m, 15–17 Jan 1977, *Steyermark et al. 112992* (GH, MO); Medio Caura, selva del Salto de Para, 300 m, 5 Mar 1939, *Williams 11385* (BM, F).

##### Discussion.

*Selaginella pubimarginata* is characterized by its moss-like habit, lateral leaf ovate-deltate, with tooth-like, short hairs on the upper surfaces along basiscopic halves of leaf laminae, long-ciliate margins along proximal ½, and acute to attenuate to shortly acuminate apices, axillary leaves similar in overall shape and apices to lateral leaves and long-ciliate margins along proximal ½.

*Selaginella pubimarginata* belongs in the “*Selaginella deltoides* group”, and among species in this alliance it is morphologically close to *S. albolineata* and *S. papillosa*. It is set aside from *S. albolineata* by characters listed in the diagnosis. *Selaginella pubimarginata* is distinct from *S. albolineata* by its axillary leaves ovate-deltate (vs. ovate-elliptic) with acute to attenuate to shortly acuminate (vs. obtuse to rounded) apices, lateral leaf upper surfaces without (vs. with many) idioblasts, and median leaf upper surfaces with a single, elongate and papillate idioblast on either the outer or inner halves of the laminae (vs. with two or three, elongate, and papillate idioblasts on the outer and inner halves of the laminae). *Selaginella pubimarginata* differs most noticeable from the also similar *S. papillosa* by its median leaf upper surfaces with a single (vs. lacking) elongate and papillate idioblasts on either the outer or inner halves of the laminae, with (vs. lacking) one or two, teeth-like hairs on mid, submarginal portion of the outer half of the laminae, and laminae comprised of rounded, sinuate-walled cells with the cell lumina including 1–5 (vs. 4–15) rounded (vs. conical) and not protruding (vs protruding or elevated) papillae, as well as apices covered by (vs. without) teeth-like, short hairs. In addition, the lateral leaf upper surfaces of *S. pubimarginata* are covered by many (vs. few) ca. 40 (vs. 3–5) short hairs or teeth-like projections along submarginal portion of basiscopic margins and on distal, apical portion (vs. concentrate on distal most portion) of apices. Finally, *S. pubimarginata* is further distinct from *S. papillosa* by its rugulate (vs. rugulate-reticulate) megaspores on proximal faces with a slightly developed (vs. with a well-developed) equatorial flange, with microstructure sparsely (vs. abundantly) covered by short- (vs. long-) echinae and distal faces slightly (vs. strongly) reticulate with open (vs. with open and closed) reticulae delimited by low (vs. high) muri.

All specimens of *S. pubimarginata* here cited were previously identified either as *S. brevifolia* or *S. brevifolia* vel aff. *Selaginella pubimarginata* is set aside from *S. brevifolia* by its median leaf ovate-orbicular to orbicular (vs. ovate) with the laminae almost as wide as long (vs. longer than wider) with outer and inner leaf halves about the same width (vs. outer leaf half frequently wider than the inner leaf half), attenuate to shortly acuminate (vs. long-aristate) apices, each acumen ⅕–¼ (vs. ⅓–½) the length of the leaf lamina, and margins obscurely hyaline or greenish (vs. conspicuously hyaline, especially the outer margin). *Selaginella pubimarginata* differs further from *S. brevifolia* by its lateral leaf upper surfaces when viewed with a dissecting scope with midribs not marked and of the same color as the laminae (vs. well-marked and straw-colored) and laminae epidermal cells inconspicuously (vs. conspicuously) rounded, and long-ciliate along proximal ½ (vs. ½–¾) margins.

#### Selaginella rostrata

Taxon classificationPlantaeSelaginellalesSelaginellaceae

Valdespino sp. nov.

E8E14314-6A9B-5743-9E20-59ABF16A71BF

urn:lsid:ipni.org:names:77211385-1

Figures 9 , 13

##### Diagnosis.

*Selaginella rostrata* differs from *S. microdonta* by its broadly ovate to ovate-deltate (vs. ovate to ovate-lanceolate) median leaves that are wider at middle (vs. at base) of the laminae, with cordate to subcordate (vs. oblique) bases, hyaline (vs. greenish to weakly hyaline) inner margins, strongly beveled (vs. plane or weakly beveled) apices in profile that are long-aristate (vs. acute to attenuate), the arista (0.06)0.1–0.2 mm long (vs. apices less than 0.05 mm long), usually tipped by one to three teeth (vs. bluntly tipped or tipped by one teeth).

##### Type.

**Venezuela.** Bolívar: Piar, Ptari-tepui, steep forested slopes at base of first line of sandstone bluff, on south-facing part, E of “Cave Rock,” 2130 m, 4 Nov 1944, *J.A. Steyermark 59836* (holotype: NY!; isotypes: BM-n.v., F!, MO!, US!).

##### Description.

*Plants* epipetric or terrestrial, ribbon-like, with leafy liverwort habit. *Stems* creeping, stramineous, 3–10 cm long, (0.1)0.18–0.34 mm diam., non-articulate, not flagelliform, non-stoloniferous, 1- or 2-branched, the lateral branches resulting from division of the main stem usually becomes arrested. *Rhizophores* axillary and subdorsal, borne throughout stems, 0.08–0.12 mm diam. *Leaves* heteromorphic throughout, chartaceous to thin-coriaceous, upper surfaces green or light-brown when old or due to dying process, lower surfaces silvery green or light-brown when old or due to dying process. *Lateral leaves* distant, ascending to slightly perpendicular to stems, broadly elliptic to broadly ovate, 0.7–2.0 × 0.4–1.4 mm; bases rounded to round-truncate, glabrous, acroscopic bases overlapping stems, basiscopic bases free from stems; acroscopic margins on upper surfaces greenish or weekly hyaline along proximal ½, otherwise greenish on distal ½, composed of quadrangular to rounded cells, basiscopic margins on upper surfaces greenish, bordered by quadrangular to rounded cells, margins on lower surfaces continuously bordered by a hyaline band comprised of idioblasts, the band 3–15 cells wide, the idioblasts elongate, straight-walled, and papillate, the papillae in a single row over each cell lumen, acroscopic margins dentate to denticulate throughout or denticulate along proximal ⅓−½ and entire distally, basiscopic margins sparsely denticulate or entire throughout; apices obtuse to rounded, occasionally tipped by a caducous short or tooth-like hair; upper surfaces glabrous, comprising rounded to irregularly, sinuate-walled, laevigate cells, without idioblasts or stomata; lower surfaces comprising elongate, slightly sinuate-walled cells, without idioblasts, with stomata in 1–3 rows along midribs. *Median leaves* slightly imbricate to distant, ascending, ovate to ovate-deltate with the inner halves of the leaf lamina ⅛−¼ wider than the outer halves, 0.6–1.5 × 0.4–1.0 mm; bases glabrous, cordate to subcordate, without auricles; margins bordered continuously by a hyaline band comprised of idioblasts, the band 2–5 cells wide, the idioblasts, elongate, straight-walled, and papillate, the papillae on one row on each cell lumen, denticulate throughout or entire along proximal ½ and denticulate along distal ½; apices keeled, long-acuminate to long-aristate, the acumen or arista denticulate, ⅓–¼ the length of the laminae, each (0.06–)0.1–0.2 mm long, usually tipped by one to three teeth; upper surfaces similar to lateral leaves upper surfaces, without idioblasts, with stomata in 1 or 2 rows restricted to distal ⅓ along the keel; lower surfaces similar to lower surfaces of lateral leaves, without idioblasts or stomata. *Axillary leaves* shape (except, occasionally oblong), size, bases, margins, apices and leaf surfaces similar to lateral leaves. *Strobili* terminal, single or dichotomous at branch tips, loosely quadrangular to slightly dorsiventral flattened, 2–12 mm long. *Sporophylls* slightly heteromorphic, broadly ovate to ovate-lanceolate (ventral sporophylls more broadly ovate and slightly shorter, dorsal sporophylls usually ovate-lanceolate and slightly larger), with a strongly developed keel along midrib, the keel glabrous, 1.0–1.3 × 0.5–0.9 mm; bases rounded; margins bordered by a hyaline band similar to that of the median leaves, dentate; *dorsal sporophylls* spreading, strongly keeled along midribs and especially near apices, the keel glabrous; apices acute to short-acuminate and beveled in profile and abruptly ending in a short, tooth-like cilia, the cilia often caducous; upper surfaces green and cells as in median leaves, except for the half that overlaps the ventral sporophylls where the surfaces are hyaline composed of idioblasts similar to those of the median leaves margins, lower surfaces hyaline and comprising elongate, sinuate-walled cells; *ventral sporophylls* ascending, slightly keeled along midribs, the keel glabrous; apices acute to short-acuminate, not beveled in profile and usually ending in a short cusp; upper and lower surfaces hyaline, comprised of idioblasts similar to those of the median leaves margins. *Megasporangia* along two ventral rows; *megaspores* lemon yellow, 200–220 µm diam., proximal faces rugulate-reticulate with a well-developed equatorial flange, the microstructure echinate and perforate, distal faces reticulate, the reticulae open (incomplete) to closed, the microstructure granulose and perforate; *microsporangia* on two dorsal rows; *micropores* light-orange, 30–35 µm diam., proximal and distal faces gemmate, the microstructure granulose.

**Figure 13.**
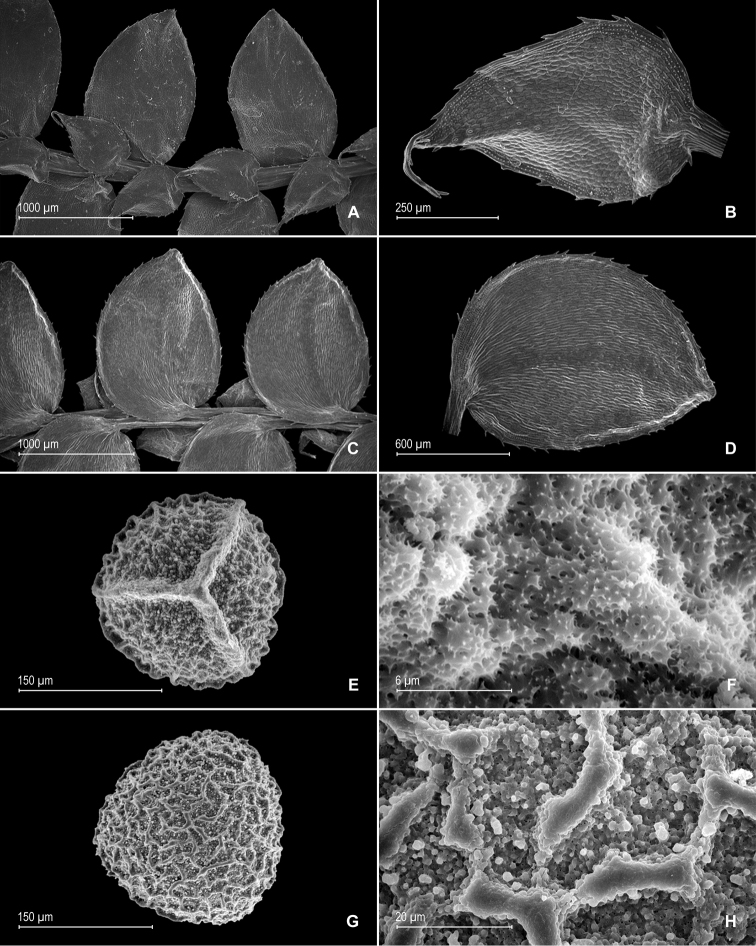
*Selaginella rostrata* Valdespino. **A** Section of upper surface of stem showing median and lateral leaves **B** median leaf, upper surface **C** section of lower surface of stem showing lateral and median leaves **D** lateral leaf, lower surface **E** megaspore, proximal face **F** detail of megaspore, proximal face **G** megaspore, distal face **H** detail of megaspore, distal face. **A–H** taken from the holotype.

##### Habitat and distribution.

*Selaginella rostrata* grow on wet and mossy sandstone bluffs, near damp stream banks, and at the base of waterfalls in mountainous tropical rainforests at 1000–2130 m. It has been found on several tepuis of the Guiana Highland in the state of Bolívar, Venezuela.

##### Etymology.

The specific epithet derives from the Latin “*rostratum*,” meaning beaked, and refers to the keeled/beaked median leaf and dorsal sporophyll apices.

##### Conservation status.

*Selaginella rostrata* is known from several collections made over a time span of more than fifty years from mid-to-late twentieth century at different localities in the Guiana Highland region of Venezuela, some of which are adjacent to the neighboring country of Guyana. This suggests an overall wide distribution. Unfortunately, the paucity of more recent collections due, in part, to a decline in botanical exploration in the aforementioned areas, does not allow for an accurate conservation assessment. Consequently, this new species is considered Data Deficient (DD) based on IUCN (2012).

##### Additional specimens examined (paratypes).

Venezuela. Bolívar: Altiplanicie of Auyan-tepui, SW sector, 05°48.02'N, 62°33.82'W, ca. 1850 m, 23–25 Apr 1996, *Anderson 13880* (MO, NY), Auyan-tepui, 1660 m, 12 May 1964, *Steyermark 93788* (US), 1820 m, 13 May 1964, *Steyermark 93871* (GH, K, NY, US), 2100 m, 17 May 1964, *Steyermark 94074* (NY, US), 1850 m, Dec 1937–Jan 1938, *Tate 1271* (NY); Cerro Venamo, 1100 m, 21 Apr 1960, *Steyermak & Nilsson 439* (NY, US-2 sheets), 1400–1450 m, 1 Jan 1964, *Steyermark et al. 92528* (GH, U, US), 1500 m, 2 Jan 1964, *Steyermark et al. 92588* (GH, U p.p., US, VEN), *Steyermark et al. 92588-A* (U p.p.), 1400–1500 m, 3 Jan 1964, *Steyermark et al. 92626* (US); Heres, Cerro Marutani, 03°50'N, 62°15'W, 1200 m, 11–14 Jan 1981, *Steyetmark et al. 123925* (GH, MO, NY), 1000–1050 m, 11–14 Jan 1981, *Steyermark et al. 124067* (GH, MO, NY); Meseta de Jáua, Cerro Jáua, 60 km NE Sanidad del Río Kanarakuni Camp, 04°45'N, 64°26'W, 1922–2100 m, 22–27 Mar 1967, *Steyermark 98018* (US), *Steyermark 98116* (US), *Steyermark 98120* (US); Piar, Amaruay-tepui, 05°54'N, 62°15'W, 700–810 m, 27 Apr 1986, *Liesner & Holst 20424 p.p.* [mixed coll. *20424a*] (MO, UC), Ptari-tepui, 2130 m, 4 Nov 1944, *Steyermark 59836* (F, MO, NY, US); Torono-tepui, Chimantá Massif, 1880–1970 m, 27 Feb 1955, *Steyermark & Wurdack 1181* (F, NY, UC, US).

##### Discussion.

*Selaginella rostrata* is characterized by its creeping, ribbon-like, leafy liverwort habit, median leaf ovate to ovate-deltate, with inner halves of leaf laminae ⅛−¼ wider than outer halves, margins continuously bordered by a hyaline band of idioblasts, each band 2–5 cells wide, the idioblasts elongate, straight-walled, and papillate, and dentate to denticulate, and apices strongly keeled, long-acuminate to long-aristate, the acumen or arista denticulate, ¼−⅓ the length of the laminae, each (0.06)0.1–0.2 mm long, each usually tipped by one to three teeth. *Selaginella rostrata* its further defined by lateral leaf broadly elliptic to broadly ovate with obtuse to rounded apices, which occasionally are tipped by a caducous short or tooth-like hair, and sporophylls (especially dorsal sporophylls) conspicuously keeled (i.e., carinate) along midribs, and with apices acute with those of dorsal sporophylls apiculate ending on a single tooth-like, short hair, and stomata along midribs and keeled apices.

*Selaginella rostrata* is morphologically close to a species group that I informally call the “*S. microdonta* group,” and in particular to the latter species and *S. neblinae*. *Selaginella rostrata* can be distinguished from *S. microdonta*, which may still need to be further circumscribed, by the characters listed in the diagnosis and its median leaf with inner halves of leaf laminae ⅛−¼ wider than outer haves (vs. inner and outer halves of leaf laminae about the same width or inner halves ⅛ narrower than outer halves). *Selaginella rostrata* further differs from *S. microdonta* by its acroscopic lateral leaf margins dentate to denticulate throughout or denticulate along the proximal ⅓−½, and entire distally, and basiscopic margins sparsely denticulate or entire (vs. acroscopic and basiscopic margins serrate). Furthermore, *S. rostrata* is also set aside from *S. neblinae* by its broadly elliptic to broadly ovate (vs. ovate-lanceolate) lateral leaves, with obtuse to rounded (vs. long attenuate to acuminate) apices, each occasionally tipped by a caducous short or tooth-like hair (vs. hair or teeth absent), and median leaf apices acuminate to long-aristate, each acumen or aristae ¼–⅓ (vs. ½) the length of the laminae, and margins conspicuously hyaline (vs. greenish).

*Selaginella microdonta*, *S. rostrata*, and *S. neblinae* along with *S. breweriana*, *S. cardiophylla*, *S. hemicardia*, and *S. valdepilosa* are part of the *S. microdonta* group. The relationship of the “*S. valdepilosa* group” still needs to be ascertained by phylogenetic methods but an initial hypothesis would suggest that *S. breweriana* and *S. neblinae* seem to be sister species, whereas *S. cardiophylla* and *S. rostrata* form another putative sister alliance that in turn is sister to a *S. microdonta* and *S. hemicardia* alliance, to which probably *S. valdepilosa* also belongs to as an offshoot that is most distinct by its leaves and sporophylls densely, long-ciliate.

Interestingly and as part of this study, it was observed that branches of *S. rostrata* can become arrested at branch forks giving the species a ribbon-like, leafy liverwort resemblance. Valdespino (1992) previously reported this condition as resting branches in *S. hemicardia*. Furthermore, as a result of the undeveloped branches, rhizophores in both species are seemingly subdorsal.

Additionally, one paratype collection here cited (i.e., *Lierner & Holsts 20424* p.p. at MO and UC) represents mixed gatherings, which I identified as *a* = *S. rostrata* and *b* = *S. tuberculata*, while two other collections (*Steyermark et al. 92588* p.p. at U, and *Steyermark et al. 92588-A* p.p at U) are also mixed gatherings, which I identified as *a* = *S. rostrata* and *b* = *S. cardiophylla*). *Selaginella tuberculata* also has a leafy, liverwort habit and creeping stems and might eventually prove to be part of the “*S. microdonta* group,” but it is distinct from the rest of species here included in that alliance by its acroscopic lateral leaves margins long-ciliate along the proximal ¼−⅓ with upper surfaces puberulent along the basiscopic halves and towards the apices as well as occasionally also distally along the acroscopic halves.

Finally, to help identify species in the “*S. microdonta* group” a key is provided below, which summarizes distinguishing characters among taxa in this alliance.

### Key to the *Selaginella microdonta* group

**Table:** 

1	Apices of lateral and axillary leaves attenuate to acuminate	**2**
2	Margins of lateral and median leaf long-ciliate, the cilia on the acroscopic halves of the lateral leaves and on the basiscopic halves of the median leaves ⅓ to ½ the width of the laminae	***S. breweriana***
2'	Margins of lateral and median leaf entire, sparsely dentate or ciliolate, the teeth or cilia, when present, less than ⅛ the width of the laminae	***S. neblinae***
1'	Apices of lateral and axillary leaves broadly acute, obtuse to rounded	**3**
3	Vegetative leaves and sporophyll margins densely long-ciliate, leaf surfaces without idioblasts; median leaf apices tipped by two divergent, long hairs	***S. valdepilosa***
3'	Vegetative leaves and sporophyll margins sparsely dentate to denticulate or entire distally or, if short-ciliate, the leaf upper surfaces with conspicuous idioblasts; median leaf apices usually tipped by one to three teeth	**4**
4	Median leaf apices acute or short- to long-acuminate to long-aristate.	**5**
5	Median leaves widest towards the bases of the laminae, bases subcordate to rounded; upper surfaces of median and lateral leaves dull green and strongly wrinkled	***S. hemicardia***
5'	Median leaves widest at the middle of the laminae, bases cordate to subcordate; upper surfaces of median and lateral leaves shiny green and smooth	***S. rostrata***
4'	Median leaf apices acute to attenuate	**5**
6	Median and lateral leaves with conspicuous idioblasts on upper surfaces of the laminae; median leaf bases cordate to infrequently rounded and not auriculate	***S. cardiophylla***
6'	Median and lateral leaves lacking idioblasts on upper surfaces of laminae; median leaf inner bases oblique and not auricled and outer bases with a subventricose, small lobe or auricle	***S. microdonta***

#### Selaginella xanthoneura

Taxon classificationPlantaeSelaginellalesSelaginellaceae

Valdespino sp. nov.

6E834D0D-2D93-5733-B792-B9C3B2B77CBF

urn:lsid:ipni.org:names:77211386-1

**Figure 14.**
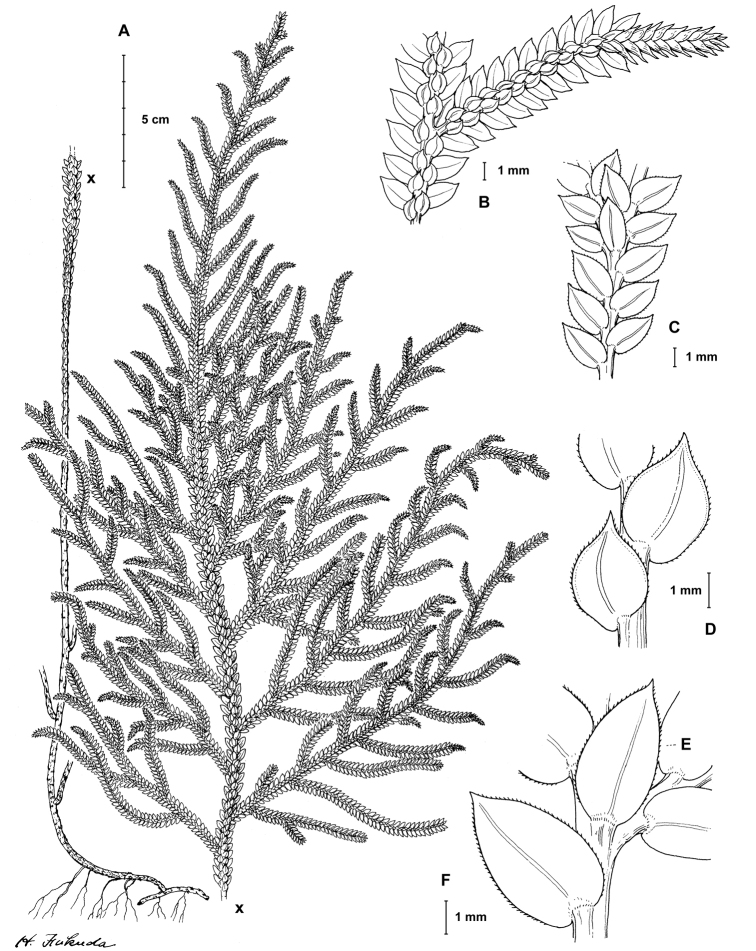
*Selaginella xanthoneura* Valdespino. **A** Habit, upper surface of stem **B** branch section, upper surface, note terminal strobilus **C** branch section, lower surface **D** branch section showing median leaves, upper surfaces **E, F** branch section showing axillary leaf (**E**) and lateral leaf (**F**), lower surfaces. **A–F** line drawing made from the holotype and isotypes as follow: **A, C–D**. **E, F** (holotype) and **B** (isotypes at GH & NY). Illustration by Haruto Fukuda.

**Figure 15.**
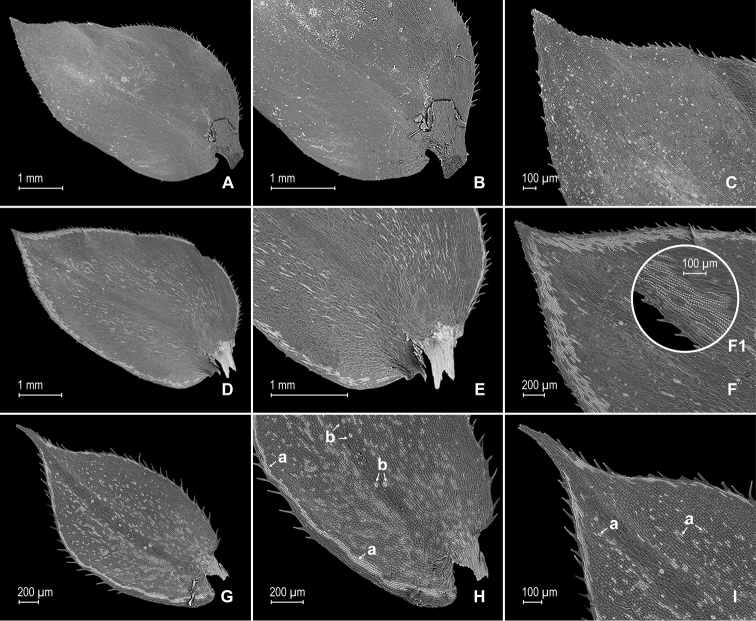
*Selaginella xanthoneura* Valdespino. **A** Lateral leaf from stem, upper surface. **B** proximal half of lateral leaf, upper surface (same leaf shown in **A**) **C** distal half of lateral leaf, upper surface (same leaf shown in **A**) **D** lateral leaf, lower surface **E** proximal half of lateral leaf, lower surface (same leaf shown in **D**) **F** distal half of lateral leaf, lower surface (same leaf shown in **D**); note, submarginal, elongate and papillate idioblasts (F1) **G** median leaf from stem branch, upper surface **H** proximal half of lateral leaf, upper surface (same leaf shown in **G**); note submarginal band of idioblasts (a) and stomata along midrib (b) **I** distal half of median leaf, upper surface (same leaf shown in **G**); note, short elongate and papillate idioblasts (a). **A–I** taken from the holotype.

**Figure 16.**
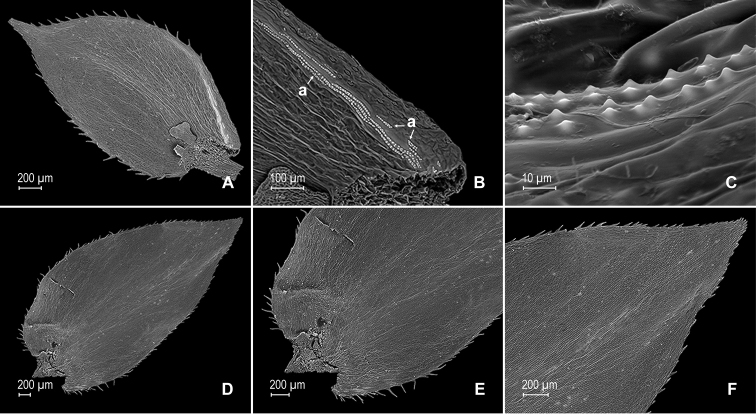
*Selaginella xanthoneura* Valdespino. **A** median leaf from stem, lower surface **B** proximal, inner half section of median leaf, lower surface (same leaf shown in **A**); note elongate and papillate idioblasts (a) **C** detail of papillae on idioblasts present on inner half section of median leaf, lower surface (same leaf shown in **A**) **D** axillary leaf from stem branch, upper surface **E** proximal half of axillary leaf, upper surface (same leaf shown in **D**) **F** distal half of axillary leaf, upper surface (same leaf shown in **D**). **A–F** taken from the holotype.

**Figure 17.**
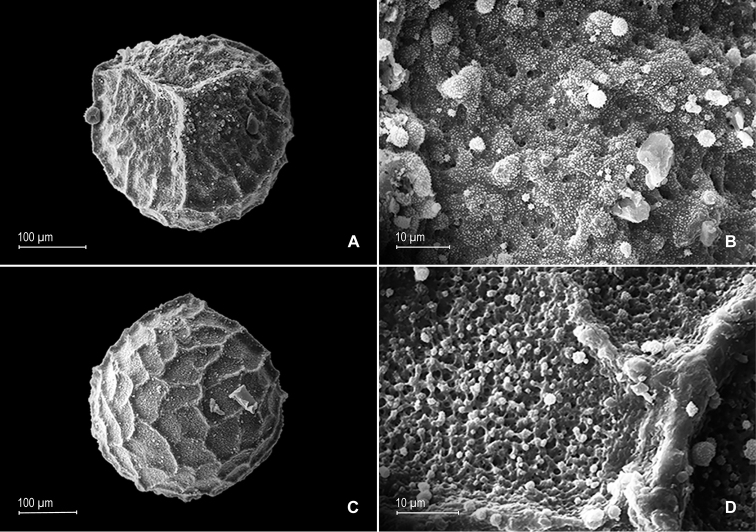
*Selaginella xanthoneura* Valdespino. **A** Megaspore, proximal face **B** detail of megaspore, proximal face **C** megaspore, distal face **D** detail of megaspore, distal face. **A–D** taken from the holotype.

**Figure 18.**
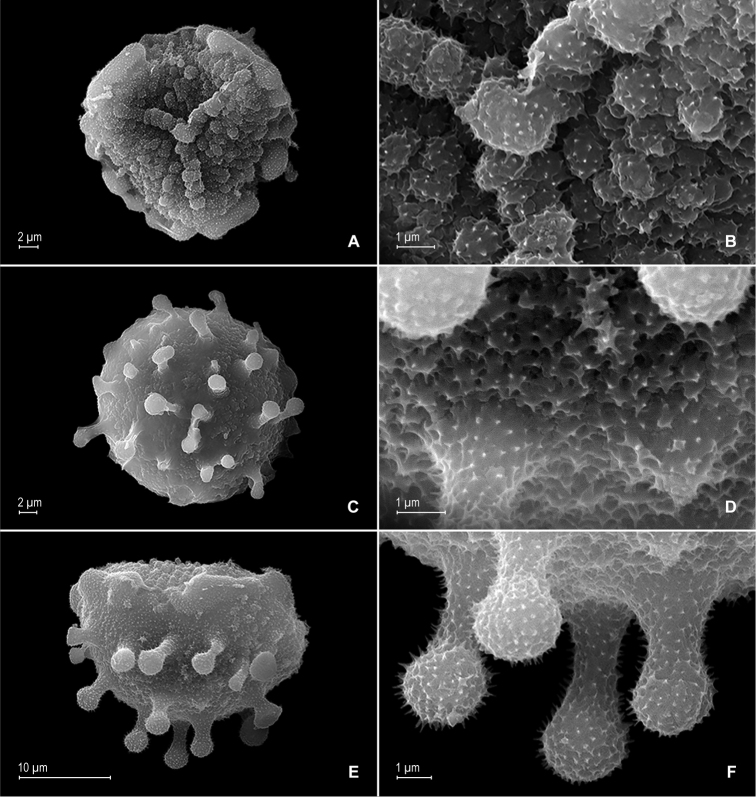
*Selaginella xanthoneura* Valdespino. **A** Microspore, proximal face **B** detail of microspore, proximal face **C** microspore, distal face **D** detail of microspore, distal face **E** microspore, distal face, equatorial view **F** detail of capitate projections with echinate microstructure surfaces. **A–F** taken from the holotype.

Figures 5 , 14 , 15 , 16 , 17 , 18

##### Diagnosis.

*Selaginella xanthoneura* is distinct from *S. hartii* Hieron. by lacking (vs. often with) flagelliform stem and branch apices, coriaceous (vs. chartaceous) leaves, lateral leaves ovate to broadly ovate (vs. ovate-oblong) with rounded (vs. truncate) bases and shortly ciliate (vs. denticulate) acroscopic margins, and acroscopic halves near proximal ⅓ of the lamina about the same width of (vs. twice as wide as) basiscopic halves, and median leaf outer margins distinctly widely hyaline (vs. green) with acuminate to short-aristate (vs. long-aristate) apices, each acumen or arista 0.1–0.3 (vs. 1.0) mm long.

##### Type.

Colombia. Magdalena [La Guajira]: Sierra de Perijá, 10 km ENE of Manaure, 46 km E of Valledupar, 3 km from the Venezuelan border, 2300 m, 4 Feb 1945 (fe), *M. Grant 10811* (holotype: COL!; isotypes: CR!, GH!, NY!, US-2 sheets!).

##### Description.

*Plants* terrestrial. *Stems* erect, stramineous, 28–60 cm tall, (0.5)1.0–2.5 mm diam. on main stem before first branches, non-articulate, not flagelliform, stoloniferous, 2 or 3-branched, the terminal portion of the stem similar in shape to lateral branches (i.e., conform). *Rhizophores* axillary, ventral, dorsal, and seemingly lateral, borne on lower-most part of the stems and throughout stolons, stout, 0.2–0.5 mm diam. *Leaves* seemingly monomorphic and strongly appressed to the stems up to shortly before (ca. 3 cm below) first branch, then heteromorphic throughout, coriaceous, upper surfaces shiny greenish yellow (i.e., citrine) when dry, smooth to slightly striate, lower surfaces shiny to silvery greenish-yellow, striate submedially and smooth towards margins, those on main stems before fully heteromorphic ovate-deltate or deltate, the bases prominently raised and truncate with both edges rounded or slightly subcordate and entire or denticulate, the margins narrowly to broadly hyaline, greenish-hyaline or greenish, inner margins short-ciliate along proximal ¼, otherwise dentate to denticulate distally, outer margins dentate to denticulate distally, apices acute, tipped by 1–4 teeth or entire. *Lateral leaves* on main stem after leaves fully heteromorphic, imbricate and ascending up to proximal ½ of the stems, otherwise distant and spreading along distal ½ of the stems, ovate to broadly ovate ascending, 4.0–6.0 × (1.5)2.0–3.5 mm; bases rounded to subcordate with a truncate and prominent central portion, glabrous, acroscopic bases strongly overlapping stems, rounded, basiscopic bases free from stems, rounded; margins on upper surfaces narrowly bordered by greenish, rectangular, and laevigate cells, acroscopic margins on lower surfaces bordered continuously by a hyaline band comprised of idioblasts, the band 2–7 cells wide, the idioblasts elongate, straight-walled, and papillate, the papillae in a single row over each cell lumen, basiscopic margins on proximal ⅓ bordered by elongate, greenish, rectangular, and laevigate cells and submarginally bordered by a hyaline band comprised of idioblasts, the band 1–5 cells wide, the idioblasts elongate, straight-walled, and papillate, the papillae in a single row over each cell lumen, on distal ⅔ bordered by a hyaline band comprised of idioblasts, the band 3–10 cells wide, the idioblasts similar to those of the acroscopic margins and submarginal proximal ⅓ of basiscopic margins and often specially toward distal ⅓ intermixed with cells similar to those of basiscopic proximal ⅓; apices acute to shortly attenuate, tipped by 1–3 teeth; upper surfaces comprising irregularly shaped, somewhat rectangular to quadrangular, sinuate-walled cells, with some sparingly distributed short, quadrangular to round, papillate idioblasts, each idioblast cell lumen with 5–8 papillae, without stomata, lower surfaces comprised of elongate, sinuate-walled cells and of elongate, straight-walled, papillate idioblasts, each idioblast cell lumen with 8–45 papillae in 1–3 rows, stomata on 1–6 rows along midribs. *Median leaves* on main stem after leaves fully heteromorphic, distant, ascending, broadly ovate, ovate-elliptic or ovate-lanceolate, 2.0–4.0 × 1.3–2.0 mm; bases glabrous, subcordate with a prominent, round outer lobe, without auricles; inner margins on upper surfaces bordered continuously by a narrowly hyaline band of idioblasts along distal ¾, the band 1–4 cells wide, the idioblasts similar to those in the hyaline marginal bands of lateral leaves acroscopic margins on lower surfaces of lateral leaves, the outer margins on upper surfaces obscurely greenish and comprising elongate, straight-walled, glabrous cells along proximal ⅓–½, on proximal ⅓–½ submargins and distal ½–⅔ margins bordered continuously by a hyaline band of idioblasts, the idioblasts similar to those in the hyaline marginal bands of lateral leaves acroscopic margins on lower surfaces but the band 2–7 cells wide and with one or two rows of papillae on cell lumen, entire along proximal ⅓ and shortly ciliate along distal ⅔; apices acuminate to short-aristate, each acumen or arista 0.1–0.3 long, tipped by 1–3 teeth; upper surfaces comprising quadrangular, rectangular to roundish, sinuate-walled cells and many, evenly distributed papillate idioblasts, each idioblasts with 5–12 papillae, stomata in 1–4 rows on distal ⅔–¾ along the midribs and marginal to submarginal along proximal ⅓ of outer margins, lower surfaces comprising elongate, straight-walled cells, without idioblasts, except for idioblasts present along proximal submarginal portion of outer margins where the idioblast are similar to those on distal ½–⅔ of outer margin on upper surfaces, without stomata. *Axillary leaves* on main stem after leaves fully heteromorphic similar in shape to lateral leaves or narrowly ovate, 3.5–4.4 × 1.5–2.4 mm; bases similar to those of lateral leaves; margins on upper and lower surfaces as those in margins of lateral leaves; apices as those of lateral leaves; both surfaces similar to those of lateral leaves. *Strobili* terminal on branch tips, quadrangular, 0.4–1.0 cm long. *Sporophylls* monomorphic, without a laminar flap, each with a slightly developed keel along midribs, the keel glabrous or with few, short, tooth-like projections distally, ovate-lanceolate, 1.4–2.0 × 0.5–1.0 mm; bases rounded; margins narrowly hyaline, 1 or 2 cells wide with the cells elongate, slightly sinuate-walled and glabrous, parallel to margins, shortly ciliate along proximal ½, denticulate on distal ½ or denticulate throughout; apices long-attenuate to long-acuminate, the acumen 0.1 or 0.2 mm long, tipped by 1 tooth; *dorsal sporophylls* with upper surfaces green and cells as in median leaves, except for the half that overlaps the ventral sporophylls where the surfaces are greenish hyaline with elongate and slightly sinuate-walled cells, lower surfaces silvery green and comprising elongate, sinuate-walled cells; *ventral sporophylls* with both surfaces, silvery green to hyaline, comprised of elongate, papillate, sinuate-walled cells. *Megasporangia* few on two proximal ventral rows; *megaspores* yellow, 100–120 µm diam., proximal faces rugulate-reticulate with a well-developed equatorial flange, the microstructure perforate and echinate, distal faces reticulate, the reticula closed, the microstructure granulate and perforate. *Microsporangia* on two dorsal rows and along most of two ventral rows; *microspores* light orange, 25–30 µm diam., proximal faces rugulate, the microstructure echinate and perforate, distal faces capitate, with each caput microechinate, the rest of the microstructure echinate and perforate.

##### Habitat and distribution.

*Selaginella xanthoneura* grows in tropical montane rainforests at 1800–2300 m. It is known from Serranía del Perijá, an extension of the eastern Andean branch (Western Cordillera) in the state of Magdalena, and in the isolated mountain range of Sierra Nevada de Santa Marta, states of César and Magdalena, both in northern Colombia, and is expected to also occur in the neighboring state of Zulia, Venezuela. It has been collected in fertile condition from February to June.

##### Etymology.

The specific epithet derives from the Gr. “*xanthos*,” yellow, and “*neuron*,” nerve, referring to the conspicuous yellow, leaf midribs on upper surfaces.

##### Conservation status.

*Selaginella xanthoneura* is known from only six collections made in two adjacent Colombian Departments, growing at high elevations, probably in and around protected areas, which however are imperiled by human encroachments and natural adversities such as landslides. It might eventually prove to be present in adjacent areas in Venezuela, but there is no current documentation to support this. It might well be that this species is relatively well protected, but the limited number of documented occurrences and distribution, as well as possible threats enumerated above indicate that it should be considered Vulnerable (VU) based on IUCN (2012).

##### Additional specimens examined (paratypes).

Colombia. **César**: Santa Marta, between Playoncito and Cuchilla Monogaca N, Pueblo Bello, 1900 m, 4 Feb 1967, *Mägdefrau 1247* (UC); Sierra Nevada de Santa Marta, Playoncito an Clisndivana? [illegible], 1800 m, Jun 1928, *Schultze 1518* (B, BM, PMA); Mpio. Manaure, Serranía del Perijá El Cinco, Finca Vistahermosa, SE de la carretera, 10°26'N, 72°57'W, 2200 m, 13 Nov 1993, *Rangel et al. 11420* (COL-image), 2235 m, 14 Nov 1993, *Pardo et al. 304* (COL-image). **Magdalena**: Sierra Neva de Santa Marta, entre San Pedro y cabeceras del Río Sevilla, slopes of La Cebolleta and Yerba Buena, ca. 2300 m and lower, 1 Feb 1959, *Barclay & Juajibioy 6808* (MO-2 sheets).

##### Discussion.

*Selaginella xanthoneura* is characterized by its fern-like habit, erect stems, each 28–60 cm tall, leaves on main stems shortly (ca. 3 cm) before first branches seemingly monomorphic and strongly appressed, the leaves after becoming fully heteromorphic with shiny, greenish yellow (i.e., citrine) upper surfaces when dry and yellowish to stramineous midribs. It is further distinguished by its median leaf bases subcordate with a prominent, round outer lobe, outer margins along proximal, submarginal ⅓–½ and distal, marginal ½–⅔ continuously bordered by a hyaline band of idioblasts, and acuminate to short-aristate apices, the upper surfaces of median leaves and the lower surfaces of lateral leaves with papillate idioblasts, and relatively short strobili, each 0.4–1.0 cm long, and with few megasporangia restricted to proximal portion of two rows of strobili ventral sporophylls.

Interestingly, one examined duplicate specimens of *S. xanthoneura* (*Schultze 1518*, B) has vegetative growth from strobili tips. This is a feature that has been amply documented in other morphologically related members of the “*Selaginella flabellata* group,” as well as on unrelated taxa from different regions of the world (Hieronymus 1901; Williams 1931; Jermy 1990; Valdespino 1993a, 1993b; Valdespino 1995; Zhang et al. 2014; Valdespino et al. 2015) and for which specific patterns were described (Valdespino et al. 2015). This same collection (*Schultze 1518*) at B and BM was originally determined by Alston as *S. hartii*, which certainly is somewhat similar to *S. xanthoneura* because of their overall fern-like habit with erect stems and leaves fully dimorphic shortly before first stem branches. *Selaginella xanthoneura* is easily set aside from the latter species by characters listed in the diagnosis. In addition, *S. xanthoneura*, at present, is only known to occur in Colombia, whereas *S. hartii* is only known from Trinidad and Tobago and the Peninsula of Paria in Sucre state, Venezuela. Furthermore, *S. xanthoneura* has a more robust plant habit with stems 2 or 3- (vs. 1 or 2 or occasionally 3-) branched.

*Selaginella xanthoneura*, as well as other members of the “*Selaginella flabellata* group,” such as *S. cheiromorpha* Alston, *S. hartwegiana* Spring, and *S. mosorongensis* Hieron. all have similar median and lateral leaf shapes. Nevertheless, *S. xanthoneura* differs from all those species by its median leaf outer bases lacking a distinct auricle and having conspicuously yellow to yellowish lateral and median leaf midribs. *Selaginella xanthoneura* is further set aside from *S. mosorongensis* by its entire (vs ciliate) median leaf outer bases.

### Emended description of *Selaginella surucucusensis*, including new distribution records and a line drawing

#### Selaginella surucucusensis

Taxon classificationPlantaeSelaginellalesSelaginellaceae

L.A. Goés & E.L.M. Assis, Kew Bull. 72: 40, 1. 2017.

2B8D7F24-1027-5F2F-878D-6AF03B86D097

Figures 5 , 19

##### Type.

Brazil. Roraima: Serra Surucucú, 26 Jan 1975, *B.G.S. Ribeiro 15.189(616)* (holotype: HRB-n.v.; isotypes: BHCB-n.v., HRB-n.v., MG!, NY!, RB!).

##### Description.

*Plants* terrestrial. *Stems* erect, stramineous, (25)35–75 cm tall, 1.2–3.0 mm diam., non-articulate, usually not flagelliform or infrequently so, stoloniferous, 3-branched, the terminal portion of the stems similar in shape to lateral branches (i.e., conform). *Rhizophores* axillary, ventral, dorsal, dorso-axillary, and seemingly lateral, borne on lower most part of stems and throughout stolons, filiform or stout, 0.2–1.0 mm diam. *Leaves* seemingly monomorphic and strongly appressed to the stem shortly before or after the first or second branches and without distinctive auricles, then heteromorphic throughout, chartaceous to coriaceous, upper surfaces shiny dark, brown-green (dark olive) to brownish (due to drying technique), seemingly smooth, lower surfaces shiny, yellowish green, dark olive to brown (due to drying technique), smooth. *Lateral leaves* on main stem after leaves become fully heteromorphic, distant, ascending to slightly spreading, ovate or ovate-oblong, 3.0–4.8 × 1.4–2.6 mm; bases rounded to truncate, glabrous, without auricles, acroscopic bases strongly overlapping stems, basiscopic bases free from stems; margins on upper and lower surfaces bordered by a narrow band comprised of greenish cells, the band 1–3 cells wide, the cells elongate, slightly sinuate-walled, and glabrous, on acroscopic margins dentate along proximal ¼, otherwise denticulate on distal ¾, on basiscopic margins entire along proximal ¾, otherwise sparsely denticulate distally; apices broadly acute to obtuse, tipped by 3–5 teeth; upper surfaces consisting of quadrangular to rectangular (jigsaw puzzle-like), sinuate-walled cells (often difficult to distinguish because of waxy deposits), many of these consisting of papillate idioblasts, comprising some sparse, elongate and papillate idioblasts, the papillae 4–11 in 1 or 2 rows on each cell lumen, without stomata; lower surfaces consisting of elongate, irregularly sinuate-walled cells (jigsaw puzzle-like) and of many elongate, straight-walled, papillate idioblasts, papillae 4–11 in 2 rows on each cell lumen, with stomata on 2–4 rows along central portion of midribs *Median leaves* on main stem after leaves fully heteromorphic, imbricate, ascending, ovate, ovate-elliptic, ovate-lanceolate to ovate-oblong, 1.7–4.2 × 0.8–2.8 mm; bases glabrous, oblique, truncate or asymmetric, inner bases rounded to truncate, outer bases auricled, the auricles ciliate with 3–14 short hairs; margins bordered continuously by a band of glabrous cells, the band 1–3 cells wide with the cells elongate, slightly sinuate-walled, glabrous, except for those on distal ⅓ of outer margins that are composed by a narrow hyaline band of idioblasts, the band 1–3 cells wide, the idioblasts straight-walled, and papillate, the papillae in one row, margins dentate to denticulate; apices acute, acuminate or aristate, each acumen or arista 0.1–0.6 mm long, tipped by 1–3 teeth; upper surfaces similar to those on upper surfaces of lateral leaves, except abundantly covered by quadrangular and elongate and papillate idioblasts, the papillae 4–32 in 1 or 2 rows on each cell lumen, with stomata in 1–5 rows along the midribs, lower surfaces comprising by elongate, irregularly sinuate-walled cells, (jigsaw puzzle-like), without idioblast and stomata. *Axillary leaves* on main stem after leaves fully heteromorphic ovate to ovate-lanceolate, 2.0–4.0 × 0.8–2.2 mm; bases rounded to slightly truncate, entire, without auricles; inner and outer margins as acroscopic margins of lateral leaves, denticulate throughout; apices broadly acute to obtuse, tipped by 1–4 teeth; both surfaces as lateral leaves. *Strobili* terminal on main stem and each branch tip, quadrangular, 0.5–4.0 cm. *Sporophylls* monomorphic, without a laminar flap, each with a slightly developed and glabrous keel along distal ¾ of the midrib, ovate to ovate-lanceolate, 0.8–1.2 × 0.4–0.7 mm; bases rounded; margins narrowly hyaline, 1 or 2 cells wide with the cells elongate, slightly sinuate-walled and glabrous, parallel to margins, denticulate throughout; apices shortly acuminate, the acumen 0.1–0.2 mm long, tipped by 1–3 teeth-like projections; *dorsal sporophylls* with upper surfaces green and cells as in median leaves, except for the half that overlaps the ventral sporophylls where the surfaces are greenish hyaline comprising elongate, slightly sinuate-walled cells, lower surfaces silvery green, comprised of elongate, sinuate-walled cells; *ventral sporophylls* with both surfaces hyaline, comprised of elongate, sinuate-walled cells and of papillate idioblasts. *Megasporangia* in two ventral rows; *megaspores* white, 240–310 µm diam., proximal faces rugulate-reticulate without an equatorial flange, the microstructure strongly echinate and perforate, distal faces reticulate the reticulae open (incomplete) to closed, each reticulum with low muri, the microstructure strongly echinate and perforate. *Microsporangia* in two dorsal rows; *microspores* orange, 23–27 µm diam., proximal faces rugulate, the microstructure echinate and granulate, distal faces capitate or baculate, the microstructure of capita or bacula and the rest of the surface echinate.

**Figure 19.**
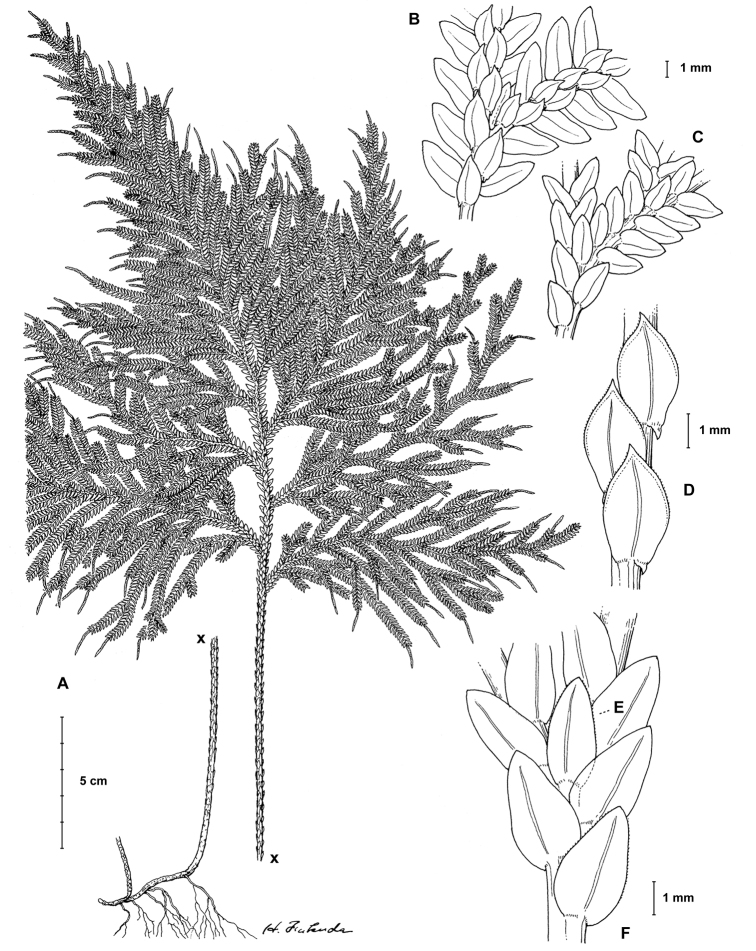
*Selaginella surucucusensis***A** Habit, upper surface of stem **B** branch section, upper surface. **C** branch section, lower surface **D** branch section showing median leaves, upper surfaces **E, F** branch section showing axillary leaf (**E**) and lateral leaf (**F**), lower surfaces. **A–F** line drawing made from the isotype at NY. Illustration by Haruto Fukuda.

**Additional specimen examined**. Colombia. **Amazonas**: Río Miritiparaná, ca. 00°30'S, 70°40'W, 700 ft [213 m], 8 May 1952, *Schultes & Cabrera 16471* (US [cited by Crabbe and Jermy (1973) as a paratype of *S. palmiformis* Alston ex Crabbe & Jermy]. **Vaupés**: Mpio. Carurú, Caño Carurú, Comunidad del Palmar, Cachivera Pacú, camino entre cachivera y sabana de Kuw (Kuvai), 01°14'47.0"N, 71°19'23.5"W, 270–430 m, 10 Sep 2013, *Rodríguez et al. 7916* (NY). Venezuela. **Amazonas**: Depto. Atabapo, sector Norte de la Sierra Parima, cuenca alta del Río Matacuni, ca 20 km NNW de Shimada-Wochi, 03°59'N, 64°41'W, 1000–1500 m, 10 Nov 1983, *Huber & Colchester 8430* (NY-2 sheets); Depto. Atures, E del Cerro Cuao, Caño Piedra, 75 km SE de Puerto Ayacucho, 05°05'N, 67°19'W, 1050 m, Sep 1989, *Fernández et al. 6113* (NY), vicinity of and upstream from damsite, N side of Río Cataniapo, 45 km SE of Puerto Ayacucho, 05°35'N, 67°15'W, 100 m, 13 May 1980, *Steyermark et al. 122394* (MO, UC); Cerro Marahuaca, 1000 m, 3 May 1949, *Maguire & Maguire Jr. 29202* (NY, US); Cerro Sipapo (Paráque), 3 km SW of Base Camp, 200 m, 8 Feb 1949, *Maguire & Politi 28814* (NY, UC, US); Comision de Frontera, ca 0.5 km below Camp 3, 02°27'24"N, 63°56'W, 20 May 1972, *Steyermark 106041* (NY); Serranía Batata, 2 km NE of Salto Colorado, Caño Colorado, 55 km SE of Puerto Ayacucho, 05°33'N, 67°08'W, 550 m, Sep 1989, *Fernández et al. 6360* (MO, NY, US). Brazil. **Roraima**: Serra dos Surucucú, NE of mission station, 02°42–47'N, 63°33–36'W, 1000–1400 m, 17 Feb 1969, *Prance et al. 9979* (F, INPA-image, NY, R, UC, US).

##### Habitat and distribution.

*Selaginella surucucusensis* grows on humid forest floors, creek- and riverbanks in lowland to montane tropical rainforests and in open scrub savanna on white sand at 200–1500 m. It was originally described from Serra dos Surucucú in the state of Roraima, Brazil. Nevertheless, its distribution range is here significantly expanded farther north- and northwestwards into the Amazon basin region to include Colombia and Venezuela. Moreover, it is here documented to be fertile from February to November.

##### Conservation status.

This species is widely distributed at low and high elevations in tropical rainforests of South America. Accordingly, it is considered of Least Concern (LC) based on IUCN (2012).

##### Discussion.

Despite the relatively recent publication of *S. surucucusensis*, with an originally limited, corroborated distribution range in Brazil provided by Góes-Neto et al. (2017), a number of specimens from Colombia and Venezuela are known and here newly documented. The study of these broader spectrum of specimens provides a better understanding of morphological characters (including mega- and microspores ornamentation features) of the species, expanded geographic circumscription, as well as of its presumed affinities. Consequently, an emended description for *S. surucucusensis* is provided, including a novel illustration.

*Selaginella surucucusensis* is characterized by its fern-like habit, non-articulate and usually not flagelliform or infrequently so, stoloniferous, 3-branched erect stems, each (25)35–75 cm tall and 1.2–3.0 mm in diam., with axillary, lateral, and dorsal to dorso-axillary rhizophores, which are borne on the lower most part of the stems and throughout stolons, each filiform or stout, 0.2–1.0 mm diam. In addition, the leaves on main stems are seemingly monomorphic and strongly appressed to stems, shortly below or above first stem branches and after this become fully heteromorphic, with median leaf upper surfaces covered with short-elongate or punctate, papillate idioblasts, and with a small or reduced, dentate outer auricle on outer bases, and lateral leaf with scattered, elongate, papillate idioblasts on lower surfaces. Furthermore, megaspores of this species are white, rugulate-reticulate on proximal faces without an equatorial flange and with strongly echinate and perforate microstructure, reticulate with open and closed reticulae formed by low muri and reticulate-granulose on distal faces with strongly echinate and perforate microstructure. Finally, microspores of this species are orange, echinate, rugulate, and granulate on proximal faces with punctate microstructure, capitate or baculate on distal faces with each caput or bacula and the rest of the surface with an echinate microstructure. In addition, the most examined specimens of *S. surucucusensis* have their leaf upper surfaces dark, brown-greenish to brownish, probably due to being fixed in alcohol.

*Selaginella surucucusensis* is morphologically somewhat similar to *S. gioiae*, from which it is set aside by the characters listed under the diagnosis and discussion of the latter. Furthermore, because of the fern-like habit and erect stems of *S. surucucusensis* most examined specimens were variously misidentified as *S. anceps* (C. Presl) C. Presl, *S. amazonica* Spring, *S. mazaruniensis* Jenm., *S. oaxacana* Spring or *S. palmiformis*. The short-elongate or punctate, papillate idioblasts on upper surfaces in median leaf of *S. surucucusensis* are somewhat similar to those of *S. cuneata* Mickel & Beitel from Mexico. *Selaginella surucucusensis* differs from *S. cuneata* by its ovate or ovate-oblong (vs. broadly ovate to ovate-orbicular) lateral leaves; median leaves bases (on main stems after first branches) oblique, truncate or asymmetric with the outer bases prominent (vs. slightly so) with (vs. without) an outer auricle, outer halves of leaf laminae at least ¼ to ½ wider (vs. twice as narrow) than inner halves, and margins of median and acroscopic margins of lateral leaves hyaline (vs. greenish).

*Selaginella surucucusensis* also appears morphologically close to *S. oaxacana* because both have median leaf with acuminate to short-aristate apices, narrowly hyaline and denticulate margins, outer basal auricles, and axillary, lateral, and dorsal rhizophores. *Selaginella surucucusensis* is set aside from *S. oaxacana* by its lateral leaf basiscopic bases rounded to adnate to the stems (vs. geniculate to auricled) and upper surfaces of the median leaves and sporophylls with short-elongate or punctate (vs. with long) idioblasts.

*Selaginella surucucusensis* differs from *S. amazonica* by its upper surfaces of leaf shiny, dark brown-green (vs. dark olive) to brownish (due to drying technique) and smooth (vs. dark brown and corrugate), with (vs. without) punctate or elongate idioblasts, median leaves above first branches ovate, ovate-elliptic, ovate-lanceolate to ovate-oblong (vs. broadly ovate to ovate-deltate) with (vs. lacking) an outer auricle, and lateral leaves shortly below or immediately above first branches ovate to ovate-oblong (vs. ovate-deltate). *Selaginella surucucusensis* is easily set aside from *S. anceps* by its leaf on main stems seemingly monomorphic and strongly appressed to the stem shortly before or after the first or second branches (vs. up to the third of fourth) branches, truncate and without (vs. with one or two, long, incurved, and ciliate) auricles. Likewise, *S. surucucusensis* is separated from *S. mazaruniensis* by its median leaf upper surfaces smooth (vs. corrugate), those above first branch with (vs. without) short-elongate or punctate, papillate idioblasts, with an outer (vs. lacking) auricle, and branches distinctly pinnate and conform (vs. usually flabelliform).

Crabbe and Jermy (1973: 141) cited one specimen here included in *S. surucucusensis* (*Schultes & Cabrera 16471*, US) as a paratype of *S. palmiformis* and Alston et al. (1981: 256) followed them. Both species are similar in having their median leaf bases with an outer, ciliate auricle. Nevertheless, in *S. palmiformis* the auricle is less prominent and covered only by 3–6 hairs. *Selaginella surucucusensis* further differs from *S. palmiformis* by its stems 3- (vs. 1- or 2-) branched, rounded when dry (vs. quadrangular) with the overall shape of proximal branches wider at base (vs. at middle) and rhombic-triangular to deltate-triangular (vs. elliptic or elliptic-lanceolate), leaves obviously heteromorphic above first or second (vs. usually at or above fourth) branches with upper surfaces having (vs. lacking conspicuous) short-elongate or punctate, and papillate idioblasts. It differs further from the latter by median leaf margins denticulate (vs. coarsely dentate), truncate (vs. often subcordate) axillary leaf bases, and ovate-lanceolate to ovate-oblong (vs. oblong) lateral leaves. *Selaginella surucucusensis* further differs from *S. palmiformis* by its median leaves on main stems after fully heteromorphic ovate, ovate-elliptic, ovate-lanceolate to ovate-oblong (vs. broadly ovate to ovate-deltate) with arcuate (vs. straight and almost central) midribs, outer halves of leaf laminae ca. ⅛ wider than inner halves (vs. both halves about the same width), and leaf bases evenly raised (vs. centrally ventricose).

*Selaginella surucucusensis* as well as *S. altheae* Valdespino are members of the “*Selaginella flabellata* group,” and have similar microspore ornamentation but they diverge on their leaf shapes. *Selaginella surucucusensis* differs from *S. altheae* by its median leaf margins denticulate (vs. inner margins short ciliate along proximal ⅔, otherwise denticulate on distal ⅓ and outer margins entire along proximal ⅓, becoming short-ciliate along medial ⅓, otherwise denticulate on distal ⅓), with (vs. without) an outer basal auricle, and lacking marginal to submarginal stomata (vs. stomata present on proximal ¼ along outer margins), and lateral leaf acroscopic margins dentate along proximal ¼, otherwise denticulate on distal ¾ (vs. long-ciliate along proximal ½–¾, otherwise short-ciliate to dentate distally).

Finally, the presence of dorsal rhizophores in *S. altheae*, *S. oaxacana*, *S. surucucusensis*, and other members of the “*Selaginella flabellata* group” might eventually prove to be a morphological character that helps define this alliance. Nevertheless, dorsal rhizophores are also found in other heterophyllous *Selaginella* species such as *S. psittacorhyncha* (Valdespino 2017b) within subg. Stachygynamdrum, where it has not been widely reported, and is characteristic of articulate species of subg. Gymnogynum s.l. (Valdespino et al. 2018; Valdespino and López 2019), subg. Lepidophyllae (Weststrand and Korall 2016), and homophyllous species classified in subg. Rupestrae (Valdespino 1993a; Weststrand and Korall 2016). Consequently, it might well be that dorsal rhizophores are underreported in subg. Stachygynandrum and of wider occurrence in *Selaginella* or perhaps this feature has originated several times in different evolutionary lineages within the genus. Accordingly, the occurrence of dorsal rhizophores within *Selaginella* warrants further morphological, anatomical, molecular, and phylogenetic studies throughout species alliances to ascertain its evolutionary implications.

## Supplementary Material

XML Treatment for Selaginella gioiae

XML Treatment for Selaginella papillosa

XML Treatment for Selaginella pubimarginata

XML Treatment for Selaginella rostrata

XML Treatment for Selaginella xanthoneura

XML Treatment for Selaginella surucucusensis
